# Supplementary material for: Longitudinal autophagy profiling of the mammalian brain reveals sustained mitophagy throughout healthy aging
Source: EMBO J. 2024 Oct 4;43(23):21. doi: 10.1038/s44318-024-00241-y (PMC11612485; doi:10.1038/s44318-024-00241-y)
Supplement: Supplementary file 1 — Appendix [file 44318_2024_241_MOESM1_ESM.pdf]

APPENDIX

Resource

Longitudinal autophagy profiling of the mammalian brain reveals sustained mitophagy throughout healthy aging

Anna Rappe, Helena A. Vihinen, Fumi Suomi, Antti J. Hassinen, Homa Ehsan, Eija S. Jokitalo and Thomas G. McWilliams

Table of contents

Appendix Figure S1. Purkinje neuron lysosomal marker validation.

Appendix Figure S2. Additional data supplement.

Appendix Figure S3. Profiling of mitolysosome and autolysosome size across the aging brain. Contains analysis from all cell-types and regions for completeness.

Appendix Figure S4. Autophagy and mitophagy trends follow identical patterns in the cell types and regions of the aging female and male CNS.

Appendix Figure S5. Quantitation of differentially acidified lysosomes across the aging brain. Contains analysis from all cell-types and regions for completeness.

Appendix Figure S1

Analysis of lysosomal markers

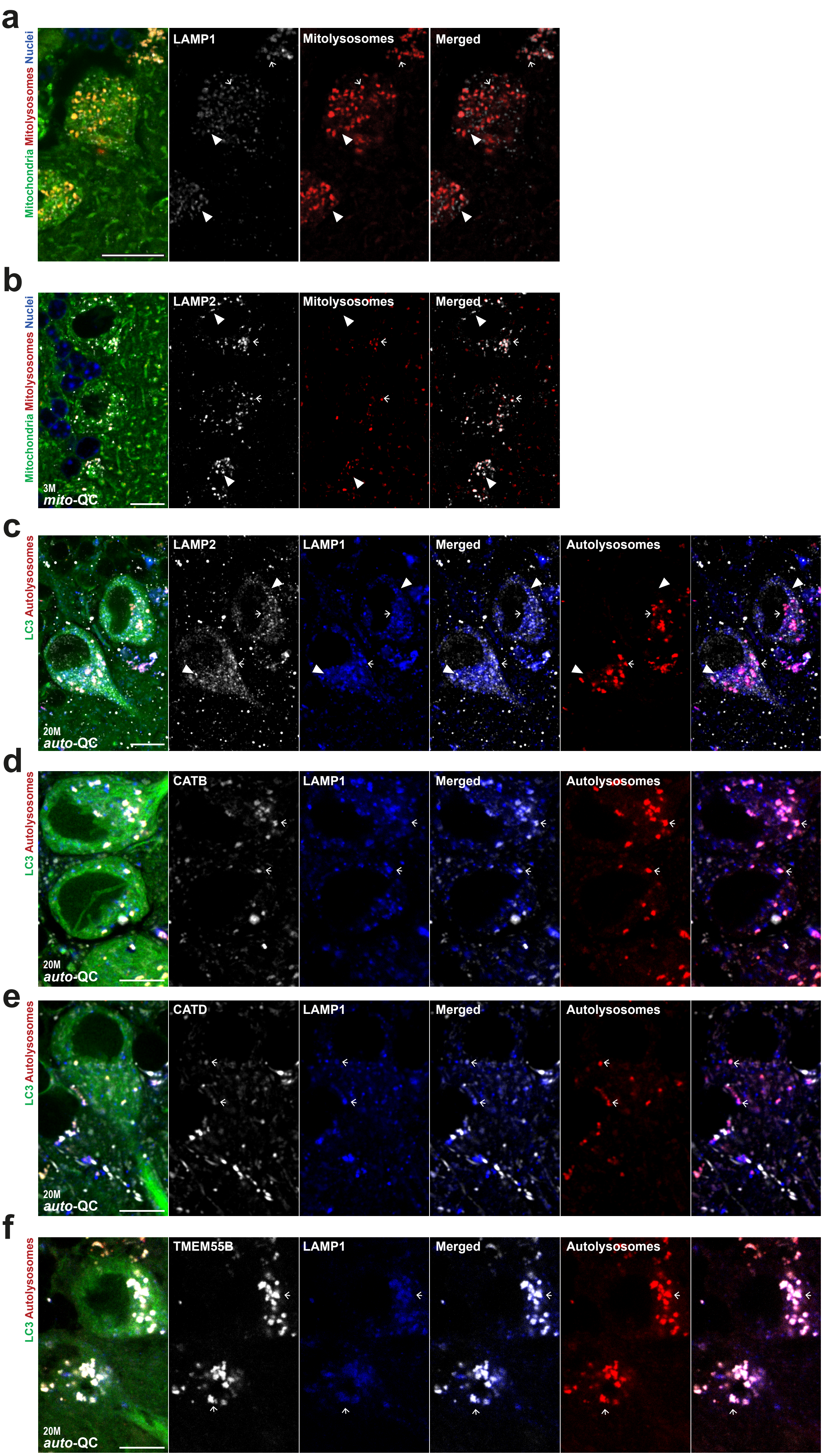

Appendix Figure S1. Purkinje neuron lysosomal marker validation.

S1a Purkinje neuron mitolysosomes are localised within LAMP1-positive endolysosomes *in vivo.* Arrowheads indicate LAMP1-only positive lysosomes while LAMP1-mCherry double-positive (mitolysosomes) puncta are indicated by arrows. 60x objective. Scale bar 20 μm.

S1b Purkinje neuron mitolysosomes are localised with LAMP2-positive endolysosomes *in vivo.* Arrowheads indicate LAMP2-only positive lysosomes while LAMP2-mCherry double-positive (mitolysosomes) puncta are indicated by arrows. 60x objective. Scale bar 10 μm.

S1c Purkinje neuron autolysosomes are localised with LAMP2-positive (gray) and LAMP1-positive (blue) endolysosomes *in vivo.* Arrowheads indicate LAMP2-LAMP1 positive lysosomes while LAMP2-LAMP1 mCherry triple-positive (autolysosomes) puncta are indicated by arrows. 100x objective. Scale bar 10 μm.

S1d Purkinje neuron autolysosomes are localised with (CATB) Cathepsin B-positive (gray) and LAMP1-positive (blue) endolysosomes *in vivo.* Arrows indicate CATB-LAMP1 mCherry triple-positive (autolysosomes) puncta.100x objective. Scale bar 10 μm.

S1e Purkinje neuron autolysosomes are localised with (CATD) Cathepsin D-positive (gray) and LAMP1-positive (blue) endolysosomes *in vivo.* Arrows indicate CATD-LAMP1 mCherry triple-positive (autolysosomes) puncta. 100x objective. Scale bar 10 μm.

S1f Purkinje neuron autolysosomes are localised with TMEM55B-positive (gray) and LAMP1-positive (blue) endolysosomes *in vivo.* Arrows indicate TMEM55B-LAMP1 mCherry triple-positive (autolysosomes) puncta are indicated by arrows. 100x objective. Scale bar 10 μm.

Appendix Figure S2

Spatial analysis - somatodendritic enrichment of mitolysosomes in cerebellar Purkinje cells

a

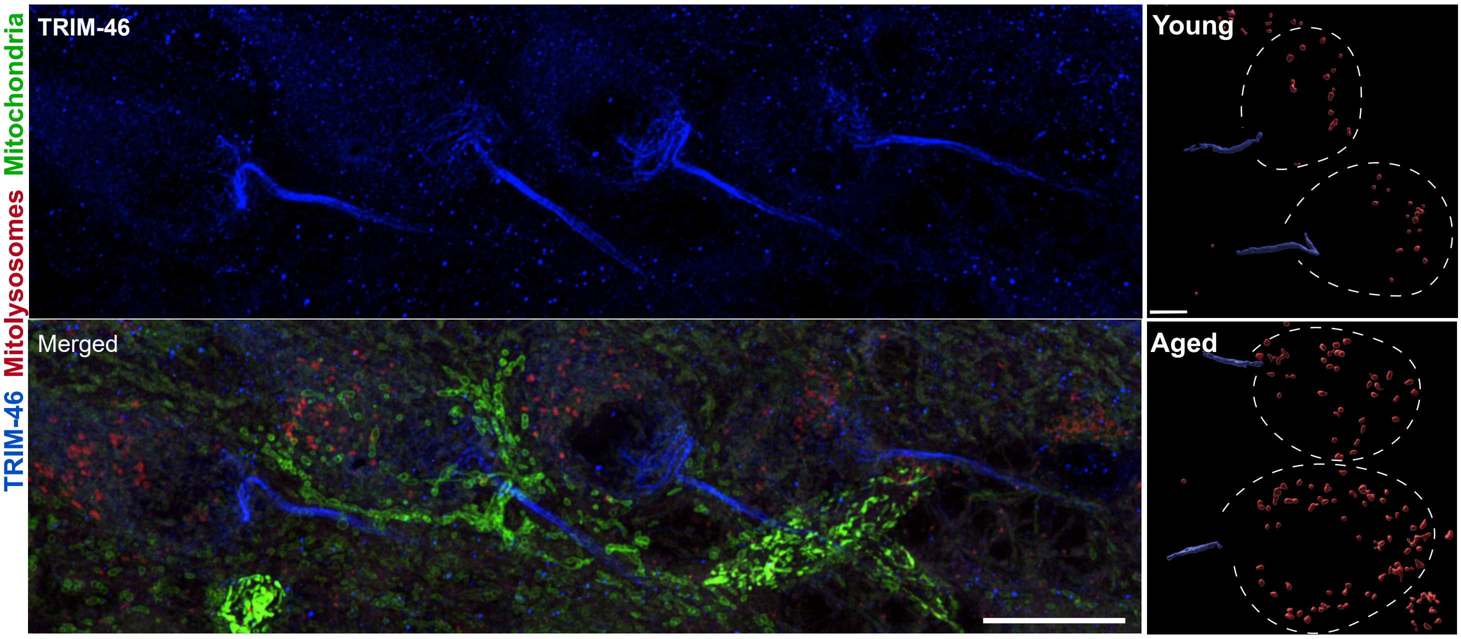

b

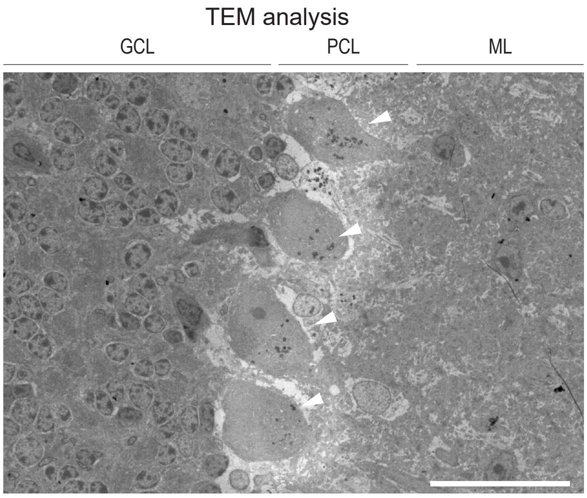

Appendix Figure S2. Additional data supplement.

S2a Representative confocal micrographs of TRIM46 immunohostochemical labelling of the axon initial segment (AIS) in Purkinje neurons, and associated 3D isosurface renders - demonstrating enrichment of mitolysosomes at the somatodendritic zones. Scale bar 20 μm.

S2b Low power TEM overview with white arrowheads highlighting electron dense lysosomes enriched at the somatodendritic compartment of Purkinje neurons *in vivo.* Scale bar 20 μm.

Appendix Figure S3

Mitolysosome and autolysosome size - all regions and cell-types

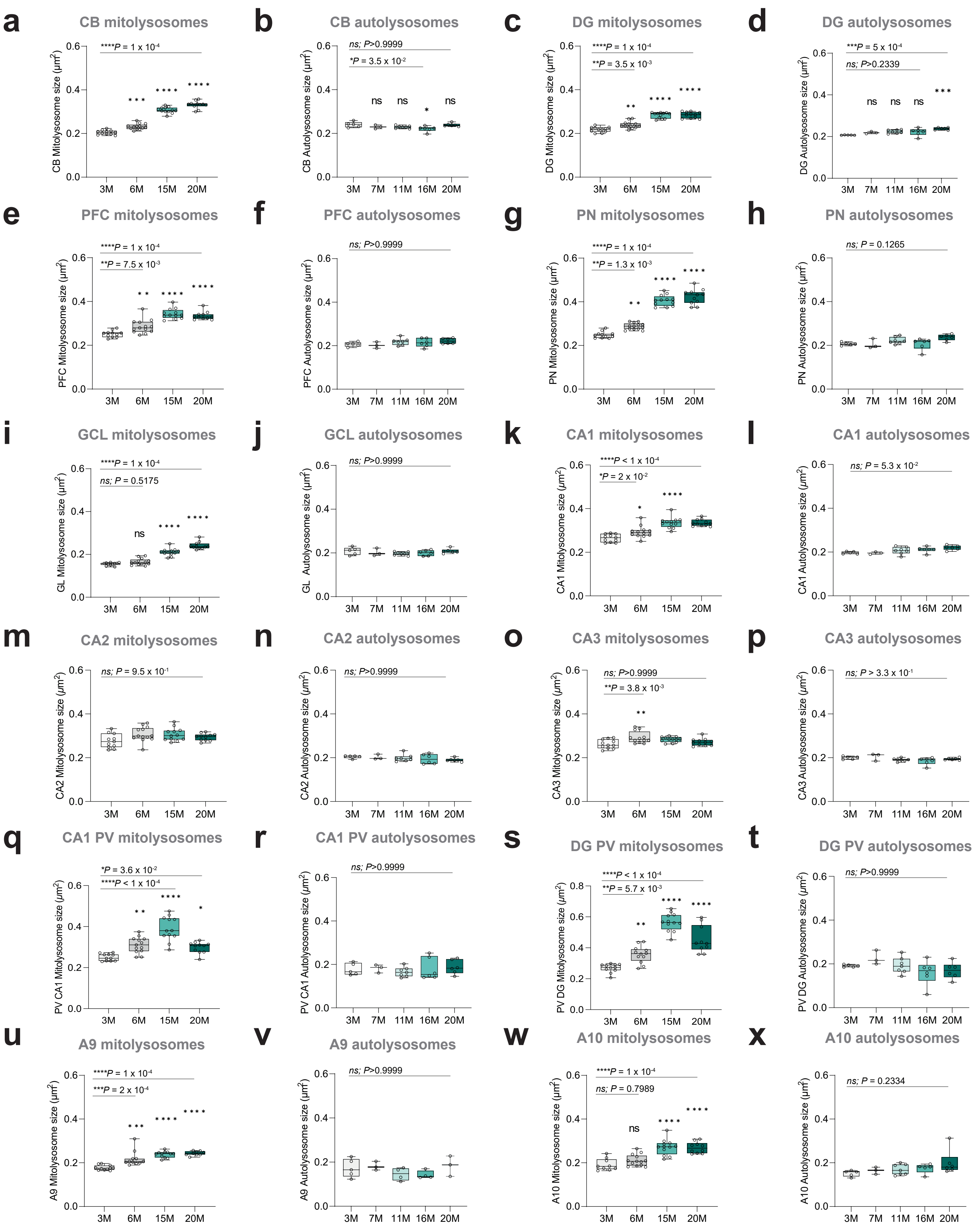

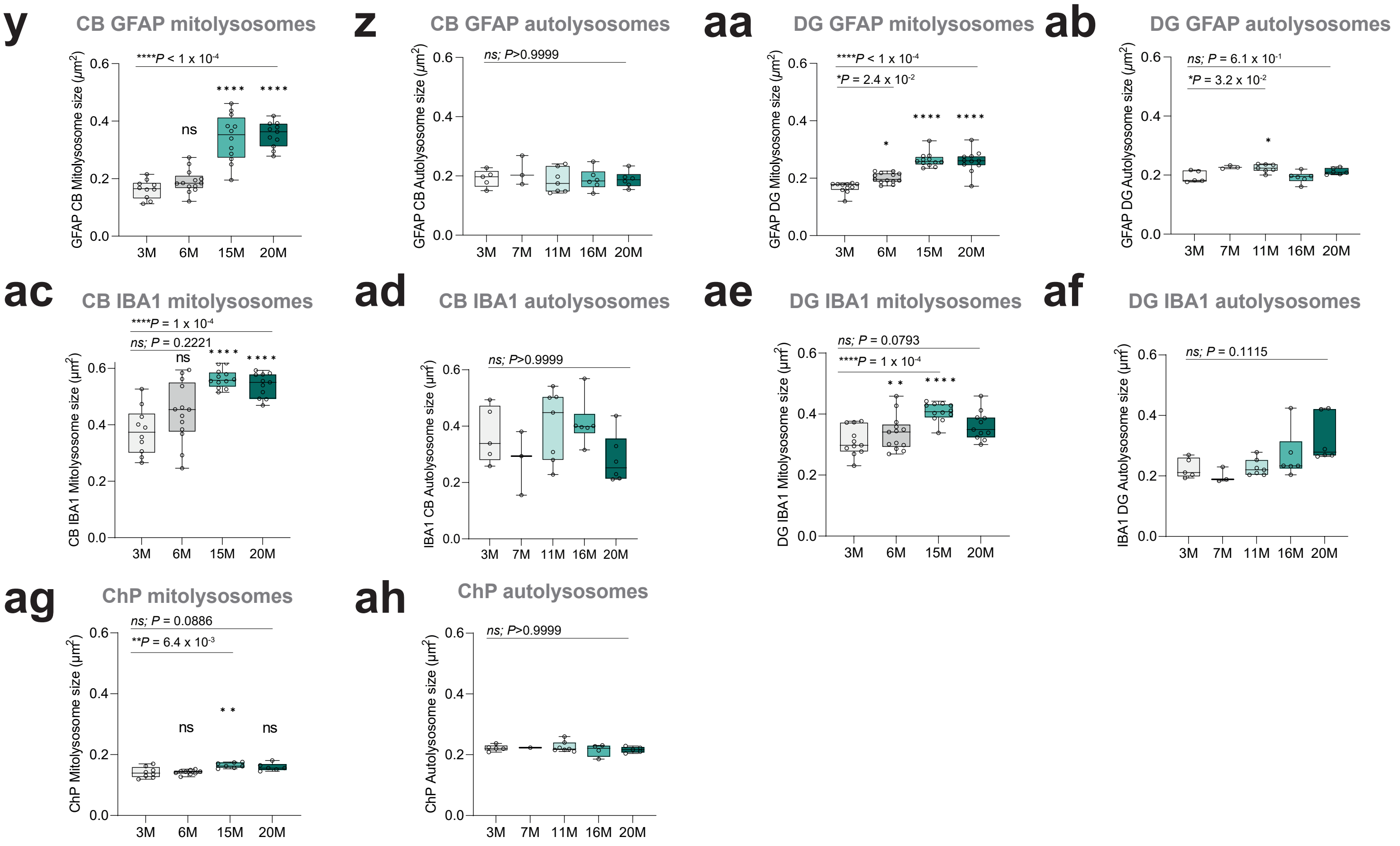
y
CB GFAP mitolysosomes
****P < 1 x 10-4
GFAP CB Mitolysosome size (μm2)
ns
3M 6M 15M 20M
z
CB GFAP autolysosomes
ns; P>0.9999
GFAP CB Autolysosome size (μm2)
3M 7M 11M 16M 20M
aa
DG GFAP mitolysosomes
****P < 1 x 10-4
*P = 2.4 x 10-2
GFAP DG Mitolysosome size (μm2)
3M 6M 15M 20M
ab
DG GFAP autolysosomes
ns; P = 6.1 x 10-1
*P = 3.2 x 10-2
GFAP DG Autolysosome size (μm2)
3M 7M 11M 16M 20M
ac
CB IBA1 mitolysosomes
****P = 1 x 10-4
ns; P = 0.2221
CB IBA1 Mitolysosome size (μm2)
ns
3M 6M 15M 20M
ad
CB IBA1 autolysosomes
ns; P>0.9999
IBA1 CB Autolysosome size (μm2)
3M 7M 11M 16M 20M
ae
DG IBA1 mitolysosomes
ns; P = 0.0793
****P = 1 x 10-4
DG IBA1 Mitolysosome size (μm2)
3M 6M 15M 20M
af
DG IBA1 autolysosomes
ns; P = 0.1115
IBA1 DG Autolysosome size (μm2)
3M 7M 11M 16M 20M
ag
ChP mitolysosomes
ns; P = 0.0886
**P = 6.4 x 10-3
ChP Mitolysosome size (μm2)
ns
ns
3M 6M 15M 20M
ah
ChP autolysosomes
ns; P>0.9999
ChP Autolysosome size (μm2)
3M 7M 11M 16M 20M

Appendix Figure S3. Profiling of mitolysosome and autolysosome size across the aging brain. Contains analysis from all cell-types and regions for completeness.

S3a — Cerebellar mean mitolysosome size. Quantitative analysis reveals increased mitolysosome size in geriatric mice compared to young. One-way ANOVA with Bonferroni *post-hoc*. ****P value <0.0001. n=47.

S3b — Cerebellar mean autolysosome size. Quantitative analysis reveals no significant changes in autolysosome size between geriatric mice compared to young. A modest decline in autolysosome size is observed at 16 months before returning to corresponding size as in young mice. One-way ANOVA with Bonferroni *post-hoc*. *P value = 0.0352, ns=not significant; P value >0.9999. n=27.

S3c — Dentate gyrus mean mitolysosome size. Quantitative analysis reveals increased mitolysosome size in geriatric mice compared to young. One-way ANOVA with Bonferroni *post-hoc*. ****P value <0.0001, **P value = 0.0035. n=47.

S3d — Dentate gyrus mean autolysosome size. Quantitative analysis reveals maintained autolysosome size throughout aging until a robust increase in size in geriatric mice compared to young. One-way ANOVA with Bonferroni *post-hoc*. ***P value = 0.0005. n=27.

S3e — PFC mean mitolysosome size. Quantitative analysis reveals increased mitolysosome size in geriatric mice compared to young. One-way ANOVA with Bonferroni *post-hoc*. ****P value <0.0001, **P value = 0.0075. n=47.

S3f — PFC mean autolysosome size. Quantitative analysis reveals no significant changes in autolysosome size between geriatric mice compared to young. One-way ANOVA with Bonferroni *post-hoc*. ns=not significant; P value >0.9999. n=27.

S3g — Purkinje cell mean mitolysosome size. Quantitative analysis reveals increased mitolysosome size in geriatric mice compared to young. One-way ANOVA with Bonferroni *post-hoc*. ****P value <0.0001, **P value = 0.0013. n=47. Also appears in Figure 3d.

S3h — Purkinje cell mean autolysosome size. Quantitative analysis reveals no significant changes in autolysosome size between geriatric mice compared to young. One-way ANOVA with Bonferroni *post-hoc*. ns=not significant; P value = 0.1265. n=27. Also appears in Figure 3d.

S3i — Granular cell layer mean mitolysosome size. Quantitative analysis reveals increased mitolysosome size in geriatric mice compared to young. One-way ANOVA with Bonferroni *post-hoc*. ****P value <0.0001. n=47.

S3j Granular cell layer mean autolysosome size. Quantitative analysis reveals no significant changes in autolysosome size between geriatric mice compared to young. One-way ANOVA with Bonferroni *post-hoc*. ns=not significant; *P* value >0.9999. n=27.

S3k CA1 mean mitolysosome size. Quantitative analysis reveals increased mitolysosome size in geriatric mice compared to young. One-way ANOVA with Bonferroni *post-hoc*. *****P* value <0.0001. **P* value = 0.0206. n=47.

S3l CA1 mean autolysosome size. Quantitative analysis reveals no significant changes in autolysosome size between geriatric mice compared to young. One-way ANOVA with Bonferroni *post-hoc*. ns=not significant; *P* value = 0.0533. n=27.

S3m CA2 mean mitolysosome size. Quantitative analysis reveals no significant change in size in geriatric mice compared to young. One-way ANOVA with Bonferroni *post-hoc*. ns=not significant; *P* value = 0.9451. n=47.

S3n CA2 mean autolysosome size. Quantitative analysis reveals no significant changes in autolysosome size between geriatric mice compared to young. One-way ANOVA with Bonferroni *post-hoc*. ns=not significant; *P* value >0.9999. n=27.

S3o CA3 mean mitolysosome size. Quantitative analysis reveals increased mitolysosome size in adult mice compared to young. In geriatric mice the size returns to corresponding values as in young. One-way ANOVA with Bonferroni *post-hoc*. ***P* value = 0.0038. ns=not significant; *P* value >0.9999. n=47.

S3p CA3 mean autolysosome size. Quantitative analysis reveals no significant changes in autolysosome size between geriatric mice compared to young. One-way ANOVA with Bonferroni *post-hoc*. ns=not significant; *P* value = 0.3287. n=27.

S3q CA1 Parvalbumin interneuron mean mitolysosome size. Quantitative analysis reveals increased mitolysosome size up until midlife compared to young mice. Mitolysosome size reduces in geriatric mice compared to midlife, yet remains significantly larger than in young. One-way ANOVA with Bonferroni *post-hoc*. *****P* value <0.0001. **P* value = 0.0356. n=47.

S3r CA1 Parvalbumin interneuron mean autolysosome size. Quantitative analysis reveals no significant changes in autolysosome size between geriatric mice compared to young. One-way ANOVA with Bonferroni *post-hoc*. ns=not significant; *P* value >0.9999. n=27.

S3s Dentate gyrus Parvalbumin interneuron mean mitolysosome size. Quantitative analysis reveals increased mitolysosome size in geriatric

mice compared to young. One-way ANOVA with Bonferroni *post-hoc*. ****P value <0.0001. **P value = 0.0057. n=46.

S3t Dentate gyrus Parvalbumin interneuron mean autolysosome size. Quantitative analysis reveals no significant changes in autolysosome size between geriatric mice compared to young. One-way ANOVA with Bonferroni *post-hoc*. ns=not significant; P value >0.9999. n=27.

S3u A9 DA neuron mean mitolysosome size. Quantitative analysis reveals increased mitolysosome size in geriatric mice compared to young. One-way ANOVA with Bonferroni *post-hoc*. ****P value <0.0001, ***P value = 0.0002. n=43

S3v A9 DA neuron mean autolysosome size. Quantitative analysis reveals no significant changes in autolysosome size between geriatric mice compared to young. One-way ANOVA with Bonferroni *post-hoc*. ns=not significant; P value >0.9999. n=19.

S3w A10 DA neuron mean mitolysosome size. Quantitative analysis reveals increased mitolysosome size in geriatric mice compared to young. One-way ANOVA with Bonferroni *post-hoc*. ****P value <0.0001. n=44.

S3x A10 DA neuron mean autolysosome size. Quantitative analysis reveals no significant changes in autolysosome size between geriatric mice compared to young. One-way ANOVA with Bonferroni *post-hoc*. ns=not significant; P value = 0.2334. n=26.

S3y Cerebellar astrocyte mean mitolysosome size. Quantitative analysis reveals increased mitolysosome size in geriatric mice compared to young. One-way ANOVA with Bonferroni *post-hoc*. ****P value <0.0001. n=46.

S3z Cerebellar astrocyte mean autolysosome size. Quantitative analysis reveals no significant changes in autolysosome size between geriatric mice compared to young. One-way ANOVA with Bonferroni *post-hoc*. ns=not significant; P value >0.9999. n=27.

S3aa Dentate gyrus astrocyte mean mitolysosome size. Quantitative analysis reveals increased mitolysosome size in geriatric mice compared to young. One-way ANOVA with Bonferroni *post-hoc*. ****P value <0.0001. *P value = 0.0239. n=47.

S3ab Dentate gyrus astrocyte mean autolysosome size. Quantitative analysis reveals no significant changes in autolysosome size between geriatric mice compared to young. A modest increase in size can be observed at midlife before returning to same size as seen in young counterparts. One-way ANOVA with Bonferroni *post-hoc*. *P value = 0.0315. ns=not significant; P value = 0.6117. n=27.

S3ac Cerebellar microglia mean mitolysosome size. Quantitative analysis reveals increased mitolysosome size in geriatric mice compared to

young. One-way ANOVA with Bonferroni *post-hoc*. ****P value <0.0001. n=46.

S3ad Cerebellar microglia mean autolysosome size. Quantitative analysis reveals no significant changes in autolysosome size between geriatric mice compared to young. One-way ANOVA with Bonferroni *post-hoc*. ns=not significant; P value >0.9999. n=27.

S3ae Dentate gyrus microglia mean mitolysosome size. Quantitative analysis reveals increased mitolysosome size during aging until midlife before a decrease size in geriatric mice, returning to same size as seen in young counterparts. One-way ANOVA with Bonferroni *post-hoc*. ****P value <0.0001, ns=not significant; P value = 0.0793. n=47.

S3af Dentate gyrus microglia mean autolysosome size. Quantitative analysis reveals no significant changes in autolysosome size between geriatric mice compared to young. One-way ANOVA with Bonferroni *post-hoc*. ns=not significant; P value = 0.1115. n=27.

S3ag ChP mean mitolysosome size. Quantitative analysis reveals no change in mitolysosome size between geriatric mice and young. Some increased size fluctuation is observed at midlife stages. One-way ANOVA with Bonferroni *post-hoc*. **P value = 0.0064, ns=not significant; P value = 0.0886. n=30.

S3ah ChP mean autolysosome size. Quantitative analysis reveals no significant changes in autolysosome size between geriatric mice compared to young. One-way ANOVA with Bonferroni *post-hoc*. ns=not significant; P value >0.9999. n=22.

Box plots extend from the 25th to the 75th percentiles, with a median line positioned inside the box. Whiskers denote the minimum and maximum values.

Appendix Figure S4

Sex-dependent differences in autophagy levels - all regions and cell-types

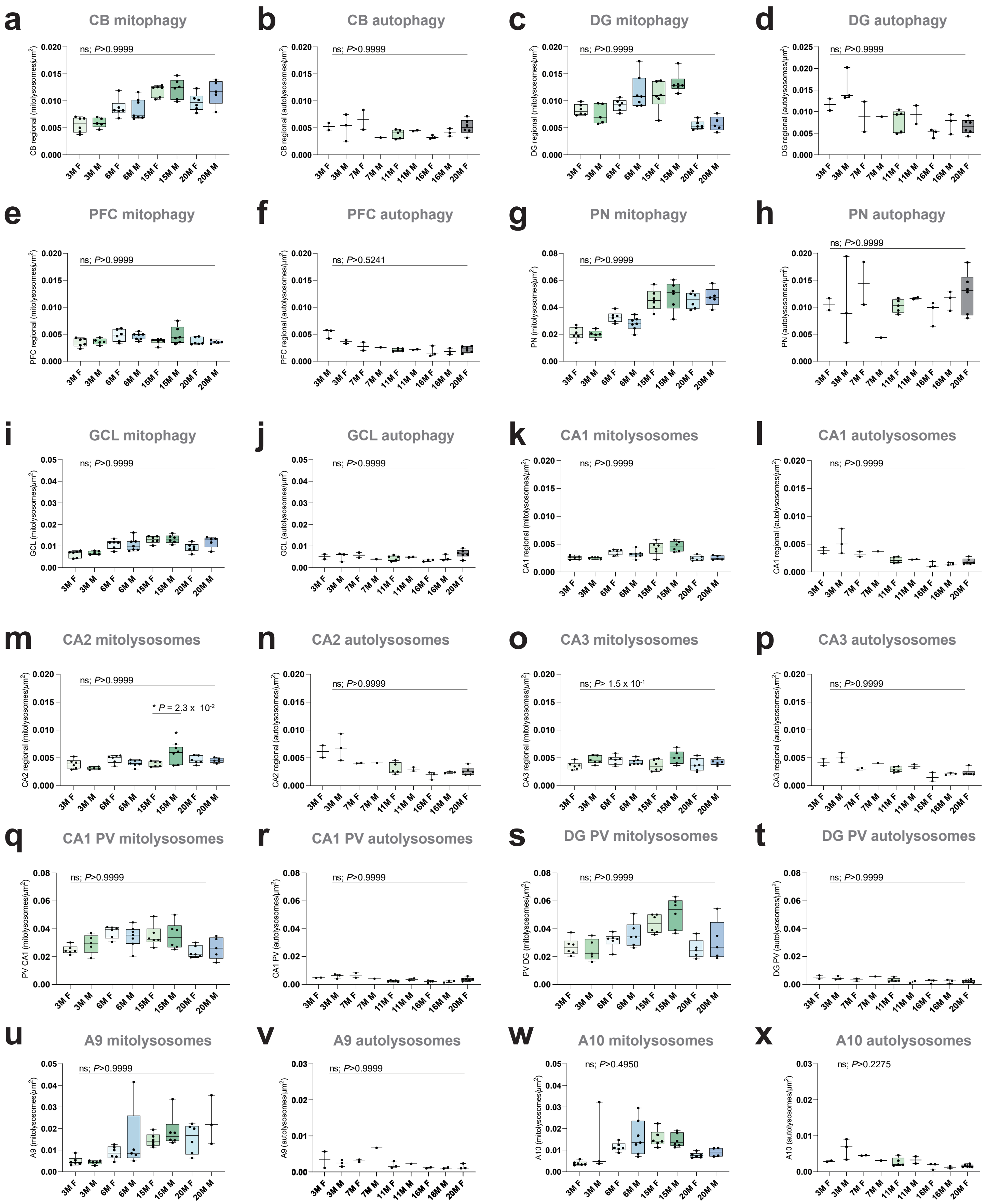

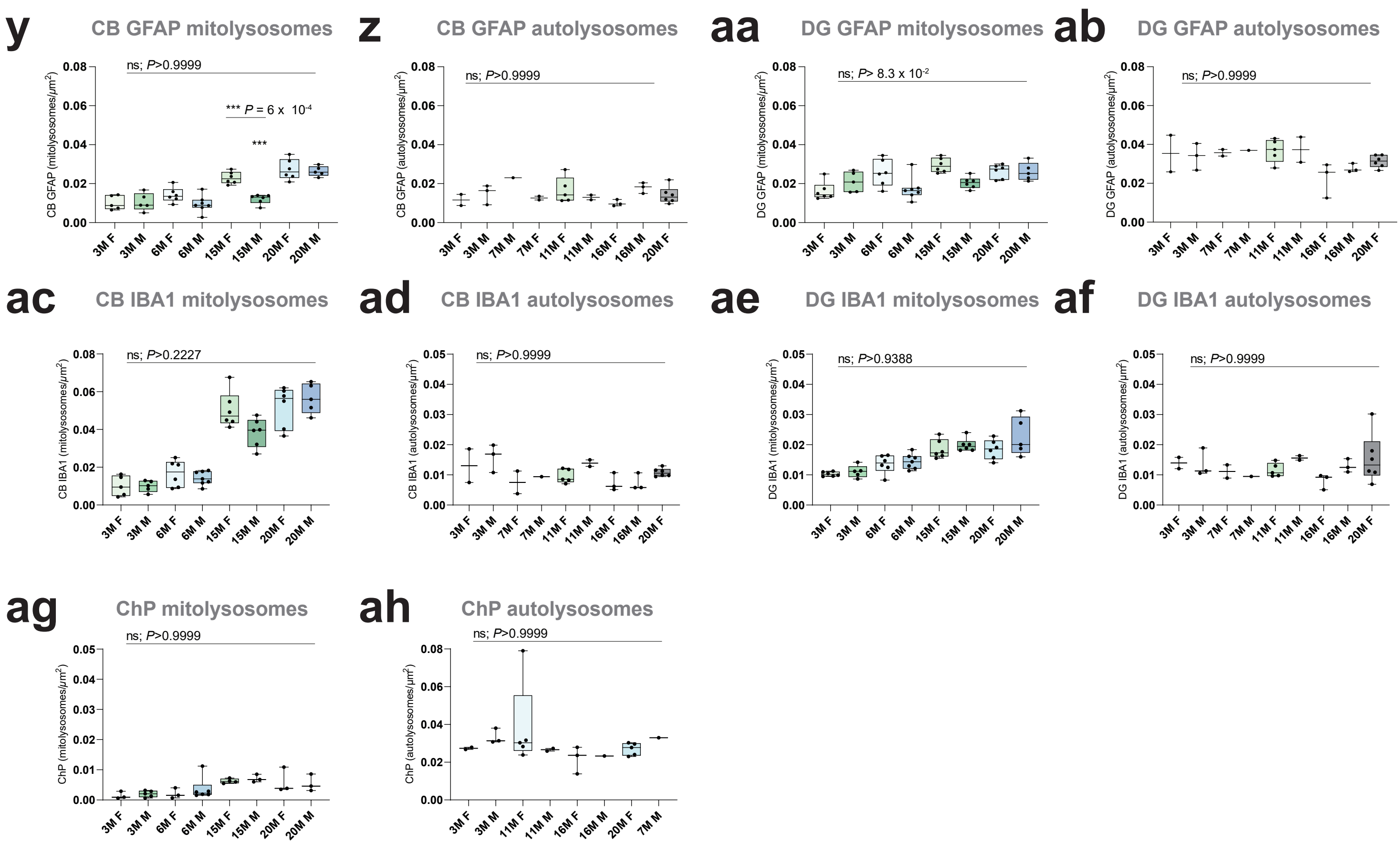
y
CB GFAP mitolysosomes
ns; P>0.9999
*** P = 6 x 10-4
***
CB GFAP (mitolysosomes/μm2)
z
CB GFAP autolysosomes
ns; P>0.9999
CB GFAP (autolysosomes/μm2)
aa
DG GFAP mitolysosomes
ns; P> 8.3 x 10-2
DG GFAP (mitolysosomes/μm2)
ab
DG GFAP autolysosomes
ns; P>0.9999
DG GFAP (autolysosomes/μm2)
ac
CB IBA1 mitolysosomes
ns; P>0.2227
CB IBA1 (mitolysosomes/μm2)
ad
CB IBA1 autolysosomes
ns; P>0.9999
CB IBA1 (autolysosomes/μm2)
ae
DG IBA1 mitolysosomes
ns; P>0.9388
DG IBA1 (mitolysosomes/μm2)
af
DG IBA1 autolysosomes
ns; P>0.9999
DG IBA1 (autolysosomes/μm2)
ag
ChP mitolysosomes
ns; P>0.9999
ChP (mitolysosomes/μm2)
ah
ChP autolysosomes
ns; P>0.9999
ChP (autolysosomes/μm2)

Appendix Figure S4. Autophagy and mitophagy trends follow identical patterns in the cell types and regions of the aging female and male CNS.

S4a Cerebellar mitophagy. Quantitative analysis reveals no significant sex-dependent differences in mitophagy levels throughout lifespan. One-way ANOVA with Bonferroni *post-hoc*. ns=not significant; *P* value >0.9999. n=47.

S4b Cerebellar autophagy. Quantitative analysis reveals no significant no significant sex-dependent differences in autophagy levels throughout lifespan. One-way ANOVA with Bonferroni *post-hoc*. ns=not significant; *P* value >0.9999. n=27.

S4c Dentate gyrus mitophagy. Quantitative analysis reveals no significant sex-dependent differences in mitophagy levels throughout lifespan. One-way ANOVA with Bonferroni *post-hoc*. ns=not significant; *P* value >0.8954. n=47.

S4d Dentate gyrus autophagy. Quantitative analysis reveals no significant no significant sex-dependent differences in autophagy levels throughout lifespan. One-way ANOVA with Bonferroni *post-hoc*. ns=not significant; *P* value >0.9999. n=27.

S4e PFC mitophagy. Quantitative analysis reveals no significant sex-dependent differences in mitophagy levels throughout lifespan. One-way ANOVA with Bonferroni *post-hoc*. ns=not significant; *P* value >0.8683. n=47.

S4f PFC autophagy. Quantitative analysis reveals no significant no significant sex-dependent differences in autophagy levels throughout lifespan. One-way ANOVA with Bonferroni *post-hoc*. ns=not significant; *P* value >0.5241. n=27.

S4g Purkinje cell mitophagy. Quantitative analysis reveals no significant sex-dependent differences in mitophagy levels throughout lifespan. One-way ANOVA with Bonferroni *post-hoc*. ns=not significant; *P* value >0.9999. n=47.

S4h Purkinje cell autophagy. Quantitative analysis reveals no significant no significant sex-dependent differences in autophagy levels throughout lifespan. One-way ANOVA with Bonferroni *post-hoc*. ns=not significant; *P* value >0.9999. n=27.

S4i Granular cell layer mitophagy. Quantitative analysis reveals no significant sex-dependent differences in mitophagy levels throughout lifespan. One-way ANOVA with Bonferroni *post-hoc*. ns=not significant; *P* value >0.9999. n=47.

S4j Granular cell layer autophagy. Quantitative analysis reveals no significant sex-dependent differences in autophagy levels throughout

lifespan. One-way ANOVA with Bonferroni *post-hoc*. ns=not significant; *P* value >0.9999. n=27.

S4k CA1 mitophagy. Quantitative analysis reveals no significant sex-dependent differences in mitophagy levels throughout lifespan. One-way ANOVA with Bonferroni *post-hoc*. ns=not significant; *P* value >0.9999. n=47.

S4l CA1 autophagy. Quantitative analysis reveals no significant sex-dependent differences in autophagy levels throughout lifespan. One-way ANOVA with Bonferroni *post-hoc*. ns=not significant; *P* value >0.9999. n=27.

S4m CA2 mitophagy. Quantitative analysis reveals a modest difference in mitophagy levels at midlife between male and female. One-way ANOVA with Bonferroni *post-hoc*. **P* value = 0.0233. ns=not significant; *P* value >0.9999. n=47.

S4n CA2 autophagy. Quantitative analysis reveals no significant sex-dependent differences in autophagy levels throughout lifespan. One-way ANOVA with Bonferroni *post-hoc*. ns=not significant; *P* value >0.9999. n=27.

S4o CA3 mitophagy. Quantitative analysis reveals no significant sex-dependent differences in mitophagy levels throughout lifespan. One-way ANOVA with Bonferroni *post-hoc*. ns=not significant; *P* value >0.1484. n=47.

S4p CA3 autophagy. Quantitative analysis reveals no significant sex-dependent differences in autophagy levels throughout lifespan. One-way ANOVA with Bonferroni *post-hoc*. ns=not significant; *P* value >0.9999. n=27.

S4q CA1 Parvalbumin interneuron mitophagy. Quantitative analysis reveals no significant sex-dependent differences in mitophagy levels throughout lifespan. One-way ANOVA with Bonferroni *post-hoc*. ns=not significant; *P* value >0.9999. n=47.

S4r CA1 Parvalbumin interneuron autophagy. Quantitative analysis reveals no significant sex-dependent differences in autophagy levels throughout lifespan. One-way ANOVA with Bonferroni *post-hoc*. ns=not significant; *P* value >0.9999. n=27.

S4s Dentate gyrus Parvalbumin mitophagy. Quantitative analysis reveals no significant sex-dependent differences in mitophagy levels throughout lifespan. One-way ANOVA with Bonferroni *post-hoc*. ns=not significant; *P* value >0.9999. n=46.

S4t Dentate gyrus Parvalbumin interneuron autophagy. Quantitative analysis reveals no significant sex-dependent differences in autophagy

levels throughout lifespan. One-way ANOVA with Bonferroni *post-hoc*. ns=not significant; *P* value >0.9999. n=27.

S4u A9 DA neuron mitophagy. Quantitative analysis reveals no significant sex-dependent differences in mitophagy levels throughout lifespan. One-way ANOVA with Bonferroni *post-hoc*. ns=not significant; *P* value >0.9999. n=43. Dentate gyrus Parvalbumin interneuron mitophagy.

S4v A9 DA neuron autophagy. Quantitative analysis reveals no significant no significant sex-dependent differences in autophagy levels throughout lifespan. One-way ANOVA with Bonferroni *post-hoc*. ns=not significant; *P* value >0.9999. n=19. Dentate gyrus Parvalbumin interneuron autophagy.

S4w A10 DA neuron mitophagy. Quantitative analysis reveals no significant sex-dependent differences in mitophagy levels throughout lifespan. One-way ANOVA with Bonferroni *post-hoc*. ns=not significant; *P* value >0.495. n=44.

S4x A10 DA neuron autophagy. Quantitative analysis reveals no significant no significant sex-dependent differences in autophagy levels throughout lifespan. One-way ANOVA with Bonferroni *post-hoc*. ns=not significant; *P* value >0.2275. n=26.

S4y Cerebellar astrocyte mitophagy. Quantitative analysis reveals a significant difference in mitophagy levels at midlife between male and female. No significant difference can be seen between male and female in the other age groups. One-way ANOVA with Bonferroni *post-hoc*. ****P* value = 0.0006. ns=not significant; *P* value >0.9999. n=46.

S4z Cerebellar astrocyte autophagy. Quantitative analysis reveals no significant sex-dependent differences in autophagy levels throughout lifespan. One-way ANOVA with Bonferroni *post-hoc*. ns=not significant; *P* value >0.9999. n=27.

S4aa Dentate gyrus astrocyte mitophagy. Quantitative analysis reveals no significant sex-dependent differences in mitophagy levels throughout lifespan. One-way ANOVA with Bonferroni *post-hoc*. ns=not significant; *P* value >0.0826. n=47.

S4ab Dentate gyrus astrocyte autophagy. Quantitative analysis reveals no significant sex-dependent differences in autophagy levels throughout lifespan. One-way ANOVA with Bonferroni *post-hoc*. ns=not significant; *P* value >0.9999. n=27.

S4ac Cerebellar microglia mitophagy. Quantitative analysis reveals no significant sex-dependent differences in mitophagy levels throughout lifespan. One-way ANOVA with Bonferroni *post-hoc*. ns=not significant; *P* value >0.2227. n=47.

S4ad Cerebellar microglia autophagy. Quantitative analysis reveals no significant no significant sex-dependent differences in autophagy levels throughout lifespan. One-way ANOVA with Bonferroni *post-hoc*. ns=not significant; P value >0.9999. n=27.

S4ae Dentate gyrus microglia mitophagy. Quantitative analysis reveals no significant sex-dependent differences in mitophagy levels throughout lifespan. One-way ANOVA with Bonferroni *post-hoc*. ns=not significant; P value >0.9388. n=47.

S4af Dentate gyrus microglia autophagy. Quantitative analysis reveals no significant no significant sex-dependent differences in autophagy levels throughout lifespan. One-way ANOVA with Bonferroni *post-hoc*. ns=not significant; P value >0.9999. n=27.

S4ag ChP mitophagy. Quantitative analysis reveals no significant sex-dependent differences in mitophagy levels throughout lifespan. One-way ANOVA with Bonferroni *post-hoc*. ns=not significant; P value >0.9999. n=30.

S4ah ChP autophagy. Quantitative analysis reveals no significant no significant sex-dependent differences in autophagy levels throughout lifespan. One-way ANOVA with Bonferroni *post-hoc*. ns=not significant; P value >0.9999. n=22.

Box plots extend from the 25th to the 75th percentiles, with a median line positioned inside the box. Whiskers denote the minimum and maximum values.

Appendix Figure S5

Differentially acidified lysosome quantitation - all regions and cell-types

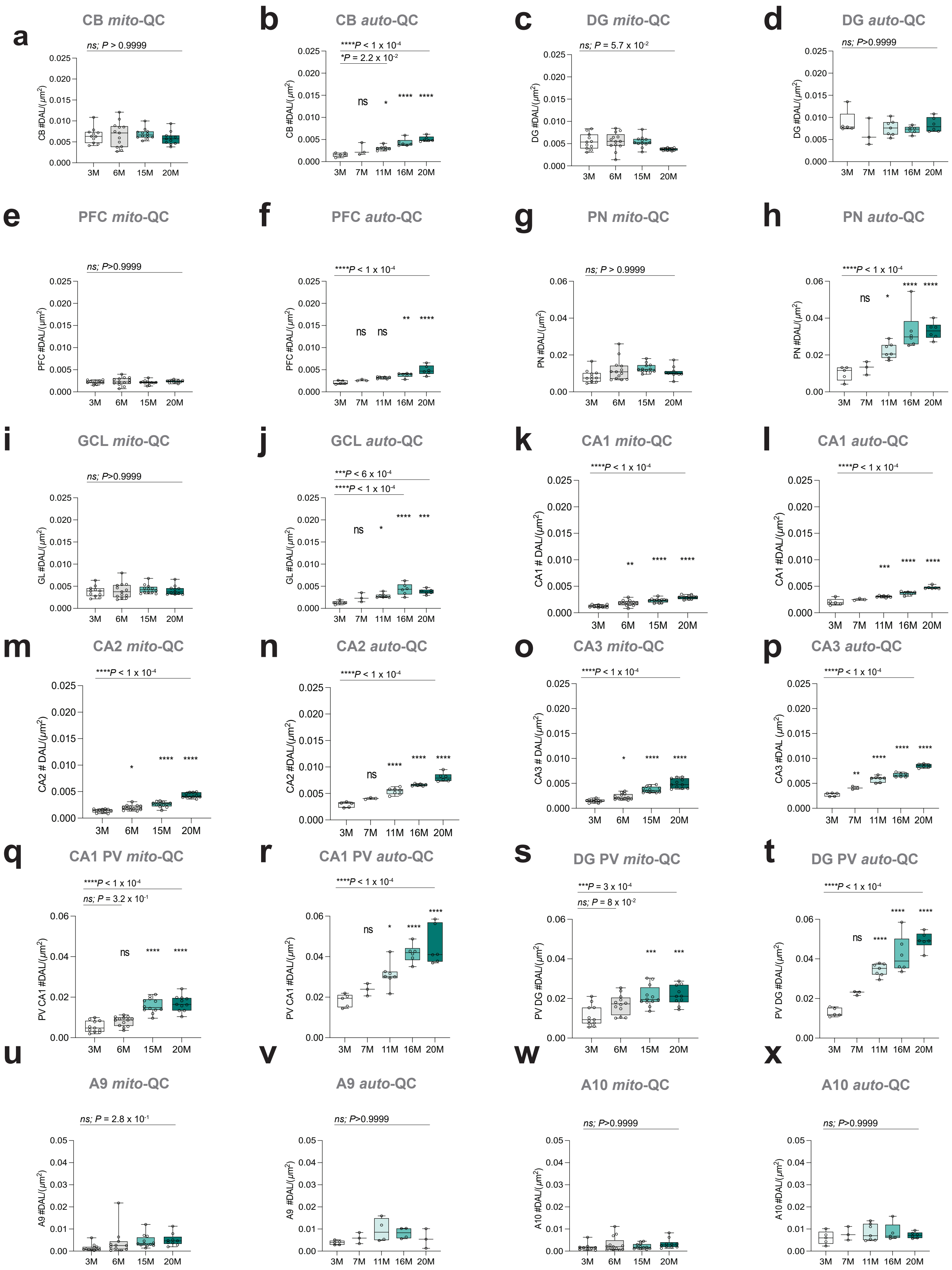

Rappe *et al.* 2024

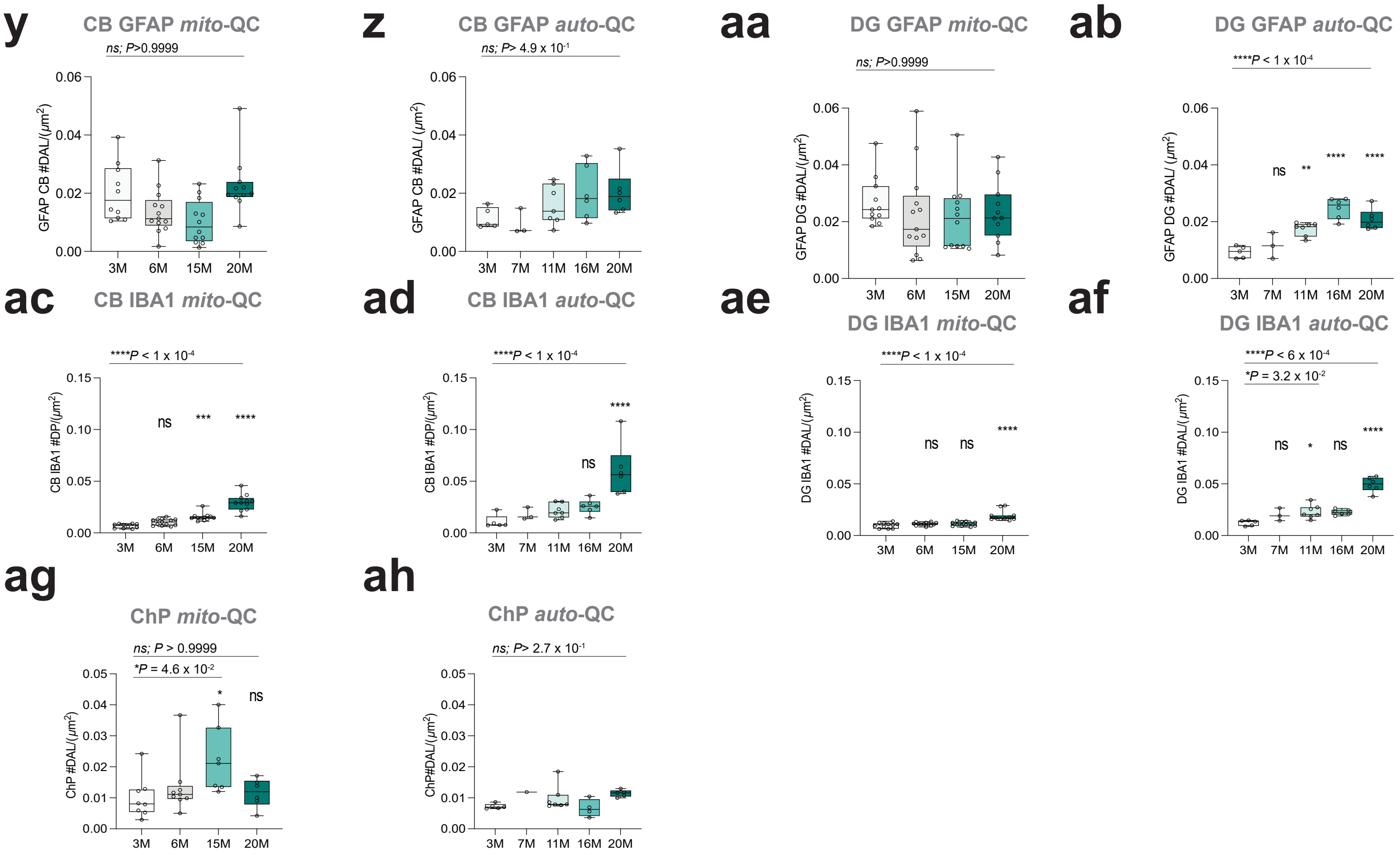
y
CB GFAP mito-QC
ns; P>0.9999
GFAP CB #DAL/(μm2)
3M 6M 15M 20M
z
CB GFAP auto-QC
ns; P> 4.9 x 10-1
GFAP CB #DAL/ (μm2)
3M 7M 11M 16M 20M
aa
DG GFAP mito-QC
ns; P>0.9999
GFAP DG #DAL/(μm2)
3M 6M 15M 20M
ab
DG GFAP auto-QC
****P < 1 x 10-4
GFAP DG #DAL/ (μm2)
ns ** **** ****
3M 7M 11M 16M 20M
ac
CB IBA1 mito-QC
****P < 1 x 10-4
CB IBA1 #DP/(μm2)
ns *** ****
3M 6M 15M 20M
ad
CB IBA1 auto-QC
****P < 1 x 10-4
CB IBA1 #DP/(μm2)
ns ****
3M 7M 11M 16M 20M
ae
DG IBA1 mito-QC
****P < 1 x 10-4
DG IBA1 #DAL/(μm2)
ns ns ****
3M 6M 15M 20M
af
DG IBA1 auto-QC
****P < 6 x 10-4
*P = 3.2 x 10-2
DG IBA1 #DAL/(μm2)
ns * ns ****
3M 7M 11M 16M 20M
ag
ChP mito-QC
ns; P > 0.9999
*P = 4.6 x 10-2
ChP #DAL/(μm2)
* ns
3M 6M 15M 20M
ah
ChP auto-QC
ns; P> 2.7 x 10-1
ChP#DAL/(μm2)
3M 7M 11M 16M 20M

Appendix Figure S5. Quantitation of differentially acidified lysosomes across the aging brain. Contains analysis from all cell-types and regions for completeness.

S5a Cerebellar differentially acidified lysosomes in *mito*-QC. Quantitative analysis reveals no significant change in differentially acidified lysosomes per area in geriatric mice compared to young. One-way ANOVA with Bonferroni *post-hoc*. ns=not significant; *P* value >0.9999. n=47.

S5b Cerebellar differentially acidified lysosomes in *auto*-QC. Quantitative analysis reveals a significant increase in differentially acidified lysosomes per area in geriatric mice compared to young. One-way ANOVA with Bonferroni *post-hoc*. ****P value < 0.0001. *P value = 0.0224. n=27.

S5c Dentate gyrus differentially acidified lysosomes in *mito*-QC. Quantitative analysis reveals no significant change in differentially acidified lysosomes per area in geriatric mice compared to young. One-way ANOVA with Bonferroni *post-hoc*. ns=not significant; *P* value = 0.0566. n=47.

S5d Dentate gyrus differentially acidified lysosomes in *auto*-QC. Quantitative analysis reveals no significant change in differentially acidified lysosomes per area in geriatric mice compared to young. One-way ANOVA with Bonferroni *post-hoc*. ns=not significant; *P* value >0.9999. n=27.

S5e PFC differentially acidified lysosomes in *mito*-QC. Quantitative analysis reveals no significant change in differentially acidified lysosomes per area in geriatric mice compared to young. One-way ANOVA with Bonferroni *post-hoc*. ns=not significant; *P* value >0.9999. n=47.

S5f PFC differentially acidified lysosomes in *auto*-QC. Quantitative analysis reveals a significant increase in differentially acidified lysosomes per area in geriatric mice compared to young. One-way ANOVA with Bonferroni *post-hoc*. ****P value < 0.0001. **P value = 0.0017. n=27.

S5g Purkinje cell differentially acidified lysosomes in *mito*-QC. Quantitative analysis reveals no significant change in differentially acidified lysosomes per area in geriatric mice compared to young. One-way ANOVA with Bonferroni *post-hoc*. ns=not significant; *P* value >0.9999. n=47.

S5h Purkinje cell differentially acidified lysosomes in *auto*-QC. Quantitative analysis reveals a significant increase in differentially acidified lysosomes per area in geriatric mice compared to young. One-way ANOVA with Bonferroni *post-hoc*. ****P value < 0.0001. *P value = 0.0387. n=27.

S5i Granular cell layer cell differentially acidified lysosomes in *mito*-QC. Quantitative analysis reveals no significant change in differentially acidified lysosomes per area in geriatric mice compared to young. One-way ANOVA with Bonferroni *post-hoc*. ns=not significant; *P* value >0.9999. n=47.

S5j Granular cell layer differentially acidified lysosomes in *auto*-QC. Quantitative analysis reveals a significant increase in differentially acidified lysosomes per area in geriatric mice compared to young. One-way ANOVA with Bonferroni *post-hoc*. ****P value < 0.0001. ***P value = 0.0006. *P value = 0.0403. n=27.

S5k CA1 differentially acidified lysosomes in *mito*-QC. Quantitative analysis reveals a significant increase in differentially acidified lysosomes per area in geriatric mice compared to young. One-way ANOVA with Bonferroni *post-hoc*. ****P value < 0.0001. **P value = 0.0033. n=47. Also appears in Figure 4b.

S5l CA1 differentially acidified lysosomes in *auto*-QC. Quantitative analysis reveals a significant increase in differentially acidified lysosomes per area in geriatric mice compared to young. One-way ANOVA with Bonferroni *post-hoc*. ****P value < 0.0001. ***P value = 0.0004. n=27. Also appears in Figure 4d.

S5m CA2 differentially acidified lysosomes in *mito*-QC. Quantitative analysis reveals a significant increase in differentially acidified lysosomes per area in geriatric mice compared to young. One-way ANOVA with Bonferroni *post-hoc*. ****P value < 0.0001. *P value = 0.0165. n=47. Also appears in Figure 4b.

S5n CA2 differentially acidified lysosomes in *auto*-QC. Quantitative analysis reveals a significant increase in differentially acidified lysosomes per area in geriatric mice compared to young. One-way ANOVA with Bonferroni *post-hoc*. ****P value < 0.0001. n=27. Also appears in Figure 4d.

S5o CA3 differentially acidified lysosomes in *mito*-QC. Quantitative analysis reveals a significant increase in differentially acidified lysosomes per area in geriatric mice compared to young. One-way ANOVA with Bonferroni *post-hoc*. ****P value < 0.0001. *P value = 0.0239. n=47. Also appears in Figure 4b.

S5p CA3 differentially acidified lysosomes in *auto*-QC. Quantitative analysis reveals a significant increase in differentially acidified lysosomes per area in geriatric mice compared to young. One-way ANOVA with Bonferroni *post-hoc*. ****P value < 0.0001. **P value = 0.0052. n=27. Also appears in Figure 4d.

S5q CA1 Parvalbumin interneuron differentially acidified lysosomes in *mito*-QC. Quantitative analysis reveals a significant increase in differentially

acidified lysosomes per area in geriatric mice compared to young. One-way ANOVA with Bonferroni *post-hoc*. ****P value < 0.0001. ns=not significant; P value = 0.3215. n=47. Also appears in Figure 4f.

S5r CA1 Parvalbumin interneuron differentially acidified lysosomes in *auto*-QC. Quantitative analysis reveals a significant increase in differentially acidified lysosomes per area in geriatric mice compared to young. One-way ANOVA with Bonferroni *post-hoc*. ****P value < 0.0001. *P value = 0.0189. n=27. Also appears in Figure 4f.

S5s Dentate gyrus Parvalbumin interneuron differentially acidified lysosomes in *mito*-QC. Quantitative analysis reveals a significant increase in differentially acidified lysosomes per area in geriatric mice compared to young. One-way ANOVA with Bonferroni *post-hoc*. ***P value = 0.0003. ns=not significant; P value = 0.0801. n=46. Also appears in Figure 4h.

S5t Dentate gyrus Parvalbumin interneuron differentially acidified lysosomes in *auto*-QC. Quantitative analysis reveals a significant increase in differentially acidified lysosomes per area in geriatric mice compared to young. One-way ANOVA with Bonferroni *post-hoc*. ****P value < 0.0001. n=27. Also appears in Figure 4h.

S5u A9 DA differentially acidified lysosomes in *mito*-QC. Quantitative analysis reveals no significant change in differentially acidified lysosomes per area in geriatric mice compared to young. One-way ANOVA with Bonferroni *post-hoc*. ns=not significant; P value = 0.2776. n=43.

S5v A9 DA neuron differentially acidified lysosomes in *auto*-QC. Quantitative analysis reveals no significant change in differentially acidified lysosomes per area in geriatric mice compared to young. One-way ANOVA with Bonferroni *post-hoc*. ns=not significant; P value >0.9999. n=19.

S5w A10 DA neuron differentially acidified lysosomes in *mito*-QC. Quantitative analysis reveals no significant change in differentially acidified lysosomes per area in geriatric mice compared to young. One-way ANOVA with Bonferroni *post-hoc*. ns=not significant; P value >0.9999. n=44.

S5x A10 DA neuron differentially acidified lysosomes in *auto*-QC. Quantitative analysis reveals no significant change in differentially acidified lysosomes per area in geriatric mice compared to young. One-way ANOVA with Bonferroni *post-hoc*. ns=not significant; P value >0.9999. n=26.

S5y Cerebellar astrocyte differentially acidified lysosomes in *mito*-QC. Quantitative analysis reveals no significant change in differentially acidified lysosomes per area in geriatric mice compared to young. One-

way ANOVA with Bonferroni *post-hoc*. ns=not significant; *P* value >0.9999. n= 46.

S5z Cerebellar astrocyte differentially acidified lysosomes in *auto*-QC. Quantitative analysis reveals no significant change in differentially acidified lysosomes per area in geriatric mice compared to young. One-way ANOVA with Bonferroni *post-hoc*. ns=not significant; *P* value >0.4918. n=27.

S5aa Dentate gyrus astrocyte differentially acidified lysosomes in *mito*-QC. Quantitative analysis reveals no significant change in differentially acidified lysosomes per area in geriatric mice compared to young. One-way ANOVA with Bonferroni *post-hoc*. ns=not significant; *P* value >0.9999. n= 47.

S5ab Dentate gyrus astrocyte differentially acidified lysosomes in *auto*-QC. Quantitative analysis reveals a significant increase in differentially acidified lysosomes per area in geriatric mice compared to young. One-way ANOVA with Bonferroni *post-hoc*. ****P value < 0.0001. **P value = 0.0025. n=27.

S5ac Cerebellar microglia differentially acidified lysosomes in *mito*-QC. Quantitative analysis reveals a significant increase in differentially acidified lysosomes per area in geriatric mice compared to young. One-way ANOVA with Bonferroni *post-hoc*. ****P value < 0.0001. ***P value = 0.0006. n=46.

S5ad Cerebellar microglia differentially acidified lysosomes in *auto*-QC. Quantitative analysis reveals a significant increase in differentially acidified lysosomes per area in geriatric mice compared to young. Up until midlife differentially acidified lysosomes remain at the same level as their young counterparts. One-way ANOVA with Bonferroni *post-hoc*. ****P value < 0.0001. n=27.

S5ae Dentate gyrus microglia differentially acidified lysosomes in *mito*-QC. Quantitative analysis reveals a significant increase in differentially acidified lysosomes per area in geriatric mice compared to young. Up until midlife differentially acidified lysososmes remain at the same level as their young counterparts. One-way ANOVA with Bonferroni *post-hoc*. ****P value < 0.0001. n=47.

S5af Dentate gyrus microglia differentially acidified lysosomes in *auto*-QC. Quantitative analysis reveals a significant increase in differentially acidified lysosomes per area in geriatric mice compared to young, with modest fluctuations throughout lifespan. One-way ANOVA with Bonferroni *post-hoc*. ****P value < 0.0001. *P value = 0.0316. n=27.

S5ag ChP differentially acidified lysosomes in *mito*-QC. Quantitative analysis reveals no significant change in differentially acidified lysosomes per area in geriatric mice compared to young. A modest increase in

number can be seen at midlife. One-way ANOVA with Bonferroni *post-hoc*. *P value = 0.0464. ns=not significant; P value >0.9999. n=30.

S5ah ChP differentially acidified lysosomes in *auto*-QC. Quantitative analysis reveals no significant change in differentially acidified lysosomes per area in geriatric mice compared to young. One-way ANOVA with Bonferroni *post-hoc*. ns=not significant; P value = 0.2728. n=22.

Box plots extend from the 25th to the 75th percentiles, with a median line positioned inside the box. Whiskers denote the minimum and maximum values.
