# Supplementary material for: Longitudinal autophagy profiling of the mammalian brain reveals sustained mitophagy throughout healthy aging
Source: EMBO J. 2024 Oct 4;43(23):21. doi: 10.1038/s44318-024-00241-y (PMC11612485; doi:10.1038/s44318-024-00241-y)
Supplement: Supplementary file 11 — Expanded View Figures [file 44318_2024_241_MOESM11_ESM.pdf]

Expanded View Figures

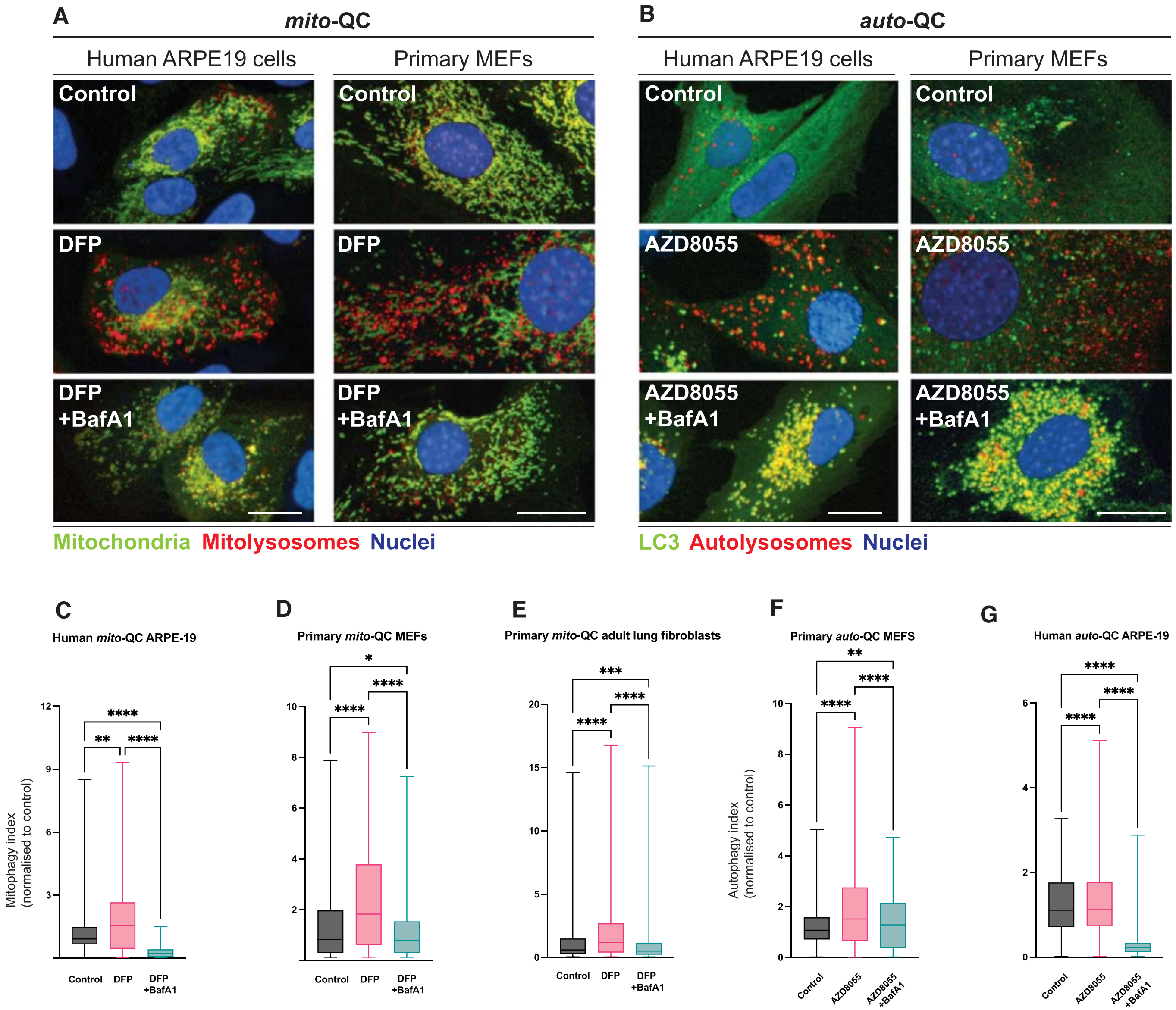

Figure EV1. Additional validation of optical reporter systems.

(**A**) Representative images of *mito*-QC human ARPE19 cells and Primary MEFs. (**B**) Representative images of *auto*-QC human ARPE19 cells and Primary MEFs. (**C**) Mitophagy in human *mito*-QC ARPE19 cells. One-way ANOVA with Tukey's post hoc. ****$P < 0.0001$, ***$P = 0.0001$. $n = 4$. (**D**) Mitophagy in *mito*-QC primary MEFS. One-way ANOVA with Tukey's post hoc. ****$P < 0.0001$. $n = 4$. (**E**) Mitophagy in adult *mito*-QC primary fibroblasts. One-way ANOVA with Tukey's post hoc. ****$P < 0.0001$, ***$P = 0.0005$. $n = 4$. (**F**) Autophagy in *auto*-QC primary MEFS. One-way ANOVA with Tukey's post hoc. ****$P < 0.0001$. $n = 4$. (**G**) Autophagy in human *auto*-QC ARPE19 cells. One-way ANOVA with Tukey's post hoc. ****$P < 0.0001$, **$P = 0.0022$. $n = 4$. Source data are available online for this figure.

© The Author(s)

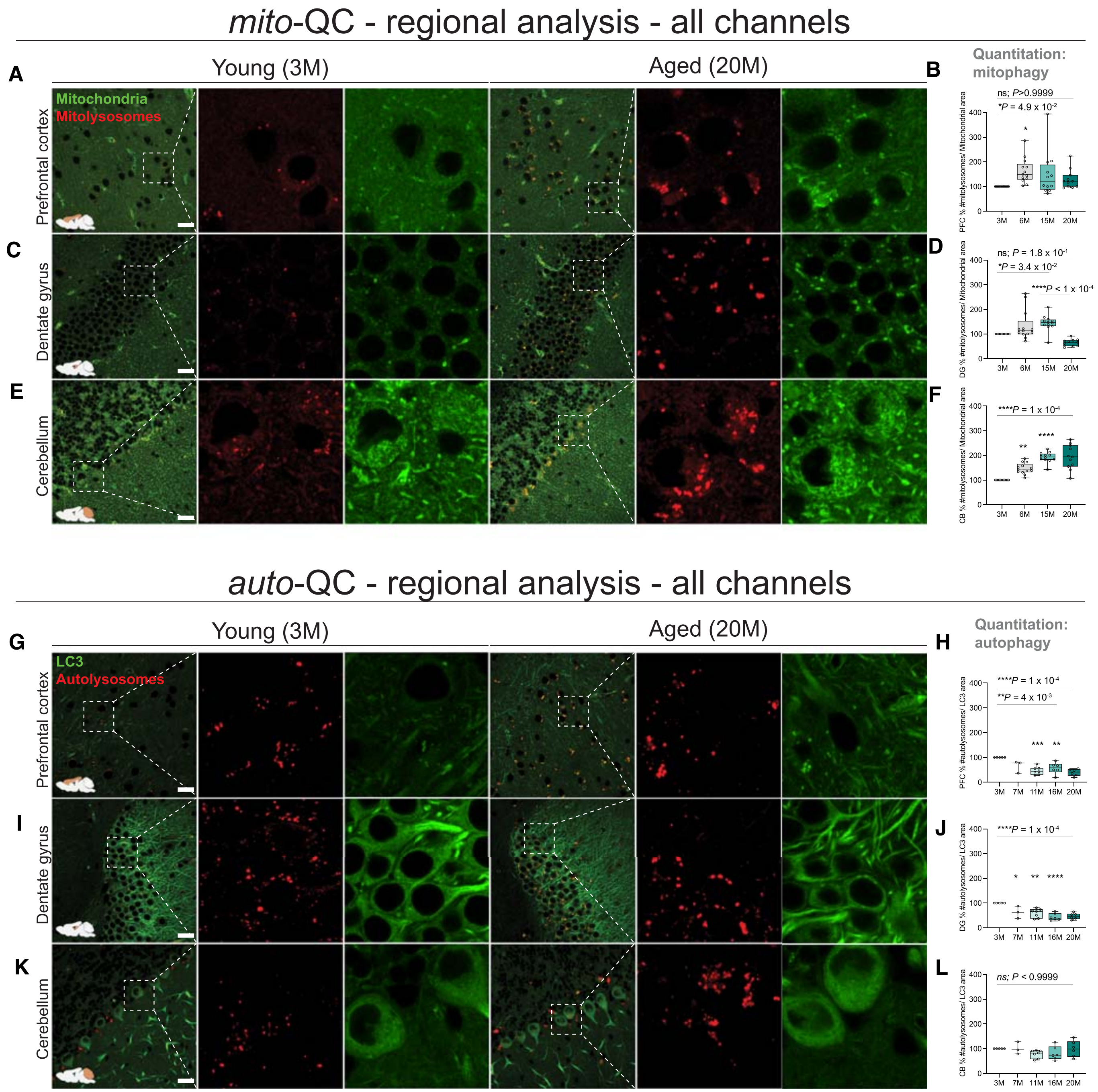
mito-QC - regional analysis - all channels
Young (3M)
Aged (20M)
A
B
C
D
E
F
Mitochondria
Mitolysosomes
Prefrontal cortex
Dentate gyrus
Cerebellum
Quantitation: mitophagy
ns; P>0.9999
*P = 4.9 x 10-2
PFC % #mitolysosomes/ Mitochondrial area
ns; P = 1.8 x 10-1
*P = 3.4 x 10-2
****P < 1 x 10-4
DG % #mitolysosomes/ Mitochondrial area
****P = 1 x 10-4
CB % #mitolysosomes/ Mitochondrial area
3M 6M 15M 20M
auto-QC - regional analysis - all channels
Young (3M)
Aged (20M)
G
H
I
J
K
L
LC3
Autolysosomes
Prefrontal cortex
Dentate gyrus
Cerebellum
Quantitation: autophagy
****P = 1 x 10-4
**P = 4 x 10-3
PFC % #autolysosomes/ LC3 area
****P = 1 x 10-4
DG % #autolysosomes/ LC3 area
ns; P < 0.9999
CB % #autolysosomes/ LC3 area
3M 7M 11M 16M 20M

 © The Author(s)

Figure EV2. Temporal dynamics of regional mitophagy and macroautophagy in the CNS—all channels (contains redisplay from Fig. 2).

(**A**, **B**) PFC mitophagy (redisplay of Fig. 2A with GFP channel). Representative confocal photomicrographs detailing instances of PFC mitophagy in young and geriatric mice. GFP and mCherry channels are shown for clarity, alongside quantitative analysis of % PFC mitophagy levels relative to mitochondrial area across all ages. Mean of 3-month group standardized to 100%. $*P = 0.0489$; ns = not significant; $P > 0.9999$. $n = 47$. (**C**, **D**) DG mitophagy (redisplay of Fig. 2C with GFP channel). Representative confocal photomicrographs detailing instances of DG mitophagy in young and geriatric mice. GFP and mCherry channels are shown for clarity, alongside quantitative analysis of % DG levels relative to mitochondrial area across all ages. Mean of 3-month group standardized to 100%. $****P < 0.0001$, $*P = 0.0338$; ns = not significant; $P = 0.1759$. $n = 47$. (**E**, **F**) CB mitophagy (redisplay of Fig. 2E with GFP channel). Representative confocal photomicrographs detailing instances of CB mitophagy in young and geriatric mice. GFP and mCherry channels are shown for clarity, alongside quantitative analysis of % CB mitophagy levels relative to mitochondrial area across all ages. Mean of 3-month group standardized to 100%. $****P < 0.0001$, $**P < 0.0014$. $n = 47$. Scale bar 20 µm. (**G**, **H**) PFC macroautophagy (redisplay of Fig. 2G with GFP channel). Representative confocal photomicrographs detailing instances of PFC macroautophagy in young and geriatric auto-QC mice. GFP and mCherry channels are shown for clarity, alongside quantitative analysis of % PFC macroautophagy levels relative to LC3 area across all ages. Mean of 3-month group standardized to 100%. $****P < 0.0001$, $***P = 0.0003$ $**P = 0.0040$. $n = 27$. (**I**, **J**) DG macroautophagy (redisplay of Fig. 2I with GFP channel). Representative confocal photomicrographs detailing instances of DG macroautophagy in young and geriatric auto-QC mice. GFP and mCherry channels are shown for clarity, alongside quantitative analysis of % DG macroautophagy levels relative to LC3 area across all ages. Mean of 3-month group standardized to 100%. $****P < 0.0001$, $**P = 0.0012$, $*P = 0.0261$. $n = 27$. (**K**, **L**) CB macroautophagy (redisplay of Fig. 2K with GFP channel). Representative confocal photomicrographs detailing instances of PFC macroautophagy in young and geriatric auto-QC mice. GFP and mCherry channels are shown for clarity, alongside quantitative analysis of % CB macroautophagy levels relative to LC3 area across all ages. Mean of 3-month group standardized to 100%. ns = not significant; $P > 0.9999$. $n = 27$. Scale bar 20 µm. Box plots extend from the 25th to the 75th percentiles, with a median line positioned inside the box. Whiskers denote the minimum and maximum values. Source data are available online for this figure.

© The Author(s)

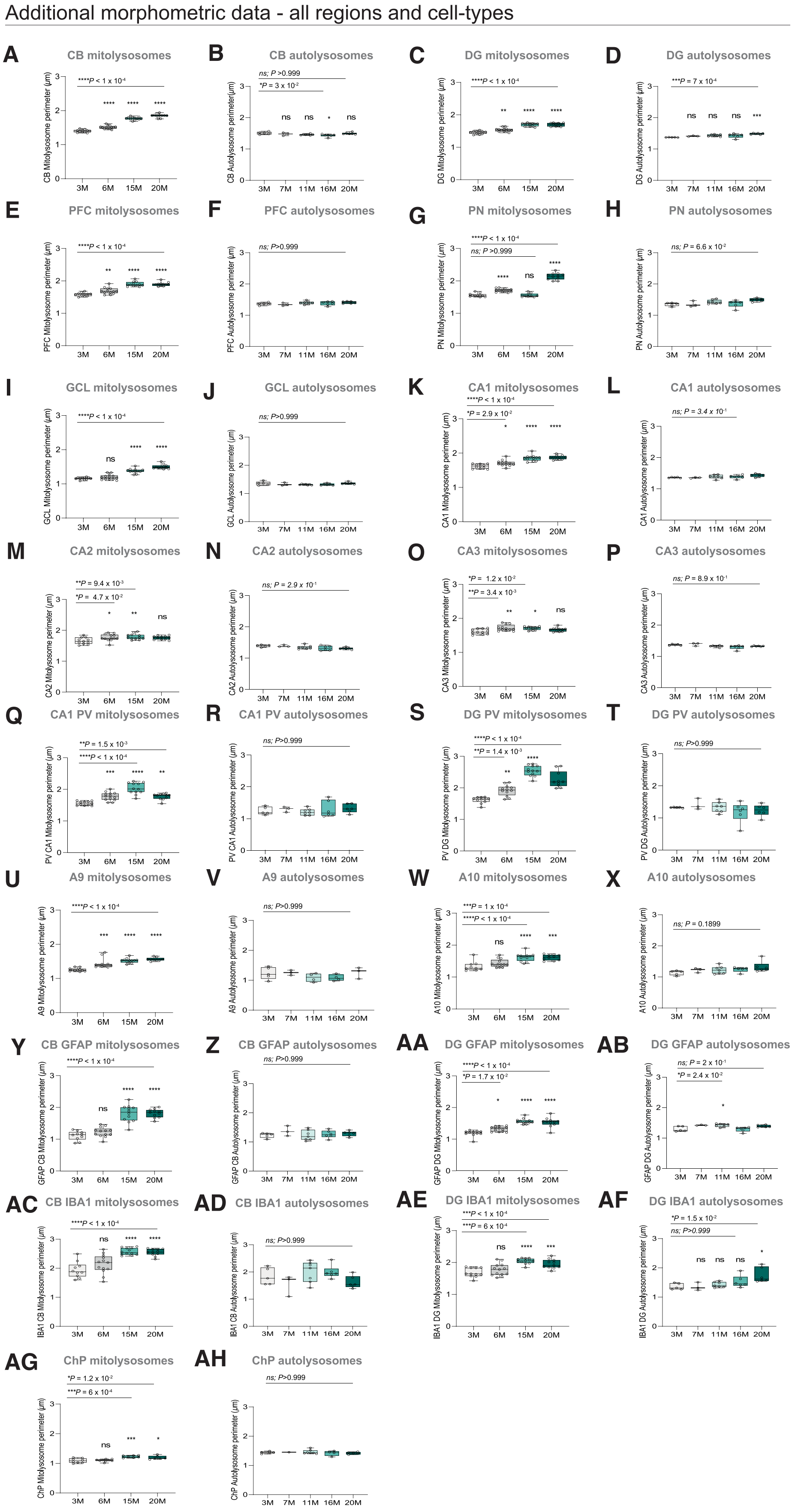
Additional morphometric data - all regions and cell-types
A CB mitolysosomes
****P < 1 x 10-4
B CB autolysosomes
ns; P >0.999
*P = 3 x 10-2
C DG mitolysosomes
****P < 1 x 10-4
D DG autolysosomes
***P = 7 x 10-4
E PFC mitolysosomes
****P < 1 x 10-4
F PFC autolysosomes
ns; P>0.999
G PN mitolysosomes
****P < 1 x 10-4
ns; P >0.999
H PN autolysosomes
ns; P = 6.6 x 10-2
I GCL mitolysosomes
****P < 1 x 10-4
J GCL autolysosomes
ns; P>0.999
K CA1 mitolysosomes
****P < 1 x 10-4
*P = 2.9 x 10-2
L CA1 autolysosomes
ns; P = 3.4 x 10-1
M CA2 mitolysosomes
**P = 9.4 x 10-3
*P = 4.7 x 10-2
N CA2 autolysosomes
ns; P = 2.9 x 10-1
O CA3 mitolysosomes
*P = 1.2 x 10-2
**P = 3.4 x 10-3
P CA3 autolysosomes
ns; P = 8.9 x 10-1
Q CA1 PV mitolysosomes
**P = 1.5 x 10-3
****P < 1 x 10-4
R CA1 PV autolysosomes
ns; P>0.999
S DG PV mitolysosomes
****P < 1 x 10-4
**P = 1.4 x 10-3
T DG PV autolysosomes
ns; P>0.999
U A9 mitolysosomes
****P < 1 x 10-4
V A9 autolysosomes
ns; P>0.999
W A10 mitolysosomes
***P = 1 x 10-4
****P < 1 x 10-4
X A10 autolysosomes
ns; P = 0.1899
Y CB GFAP mitolysosomes
****P < 1 x 10-4
Z CB GFAP autolysosomes
ns; P>0.999
AA DG GFAP mitolysosomes
****P < 1 x 10-4
*P = 1.7 x 10-2
AB DG GFAP autolysosomes
ns; P = 2 x 10-1
*P = 2.4 x 10-2
AC CB IBA1 mitolysosomes
****P < 1 x 10-4
AD CB IBA1 autolysosomes
ns; P>0.999
AE DG IBA1 mitolysosomes
***P < 1 x 10-4
***P = 6 x 10-4
AF DG IBA1 autolysosomes
*P = 1.5 x 10-2
ns; P>0.999
AG ChP mitolysosomes
*P = 1.2 x 10-2
***P = 6 x 10-4
AH ChP autolysosomes
ns; P>0.999
3M 6M 15M 20M
3M 7M 11M 16M 20M

 © The Author(s)

Figure EV3. Profiling of mitolysosome and autolysosome perimeter across the aging brain. Contains analysis from all cell-types and regions for completeness.

(**A**) Cerebellar mean mitolysosome perimeter. Quantitative analysis reveals increased mitolysosome perimeter in geriatric mice compared to young. One-way ANOVA with Bonferroni post hoc. ****$P < 0.0001$. $n = 47$. (**B**) Cerebellar mean autolysosome perimeter. Quantitative analysis reveals no significant changes in autolysosome perimeter between geriatric mice compared to young. A modest decline in autolysosome perimeter is observed at 16 months. One-way ANOVA with Bonferroni post hoc. ns = not significant; P value > 0.9999, *$P = 0.0297$. $n = 27$. (**C**) Dentate gyrus mean mitolysosome perimeter. Quantitative analysis reveals increased mitolysosome perimeter in geriatric mice compared to young. One-way ANOVA with Bonferroni post hoc. ****$P < 0.0001$. $n = 47$. (**D**) Dentate gyrus mean autolysosome perimeter. Quantitative analysis reveals a significant increase in autolysosome perimeter in geriatric mice compared to young. One-way ANOVA with Bonferroni post hoc. ***$P = 0.0007$. $n = 27$. (**E**) PFC mean mitolysosome perimeter. Quantitative analysis reveals increased mitolysosome perimeter in geriatric mice compared to young. One-way ANOVA with Bonferroni post hoc. ****$P < 0.0001$. $n = 47$. (**F**) PFC mean autolysosome perimeter. Quantitative analysis reveals no significant changes in autolysosome perimeter between geriatric mice compared to young. One-way ANOVA with Bonferroni post hoc. ns = not significant; $P > 0.9999$. $n = 27$. (**G**) Purkinje cell mean mitolysosome perimeter. Quantitative analysis reveals increased mitolysosome perimeter in geriatric mice compared to young. One-way ANOVA with Bonferroni post hoc. ****$P < 0.0001$. $n = 47$. (**H**) Purkinje cell mean autolysosome perimeter. Quantitative analysis reveals no significant changes in autolysosome perimeter between geriatric mice compared to young. One-way ANOVA with Bonferroni post hoc. ns = not significant; $P = 0.0657$. $n = 27$. (**I**) Granular cell layer mean mitolysosome perimeter. Quantitative analysis reveals increased mitolysosome perimeter in geriatric mice compared to young. One-way ANOVA with Bonferroni post hoc. ****$P < 0.0001$. $n = 47$. (**J**) Granular cell layer mean autolysosome perimeter. Quantitative analysis reveals no alterations in autolysosome perimeter between geriatric mice and young. One-way ANOVA with Bonferroni post hoc. ns = not significant; $P > 0.9999$. $n = 27$. (**K**) CA1 mean mitolysosome perimeter. Quantitative analysis reveals increased mitolysosome perimeter in geriatric mice compared to young. One-way ANOVA with Bonferroni post hoc. ****P value < 0.0001; *$P = 0.0290$. $n = 47$. (**L**) CA1 mean autolysosome perimeter. Quantitative analysis reveals no alterations in autolysosome perimeter between geriatric mice and young. One-way ANOVA with Bonferroni post hoc. ns = not significant; $P = 0.3350$. $n = 27$. (**M**) CA2 mean mitolysosome perimeter. Quantitative analysis reveals fluctuations in mitolysosome perimeter in throughout lifespan. One-way ANOVA with Bonferroni post hoc. **$P = 0.0094$, *$P = 0.0467$; ns = not significant; $P = 0.0875$. $n = 47$. (**N**) CA2 mean autolysosome perimeter. Quantitative analysis reveals no alterations in autolysosome perimeter between geriatric mice and young. One-way ANOVA with Bonferroni post hoc. ns = not significant; P value = 0.2924. $n = 27$. (**O**) CA3 mean mitolysosome perimeter. Quantitative analysis reveals fluctuations in mitolysosome perimeter in throughout lifespan. One-way ANOVA with Bonferroni post hoc. **$P = 0.0034$, *$P = 0.0122$; ns = not significant; $P = 0.2975$. $n = 47$. (**P**) CA3 mean autolysosome perimeter. Quantitative analysis reveals no alterations in autolysosome perimeter between geriatric mice and young. One-way ANOVA with Bonferroni post hoc. ns = not significant; $P = 0.8866$. $n = 27$. (**Q**) CA1 Parvalbumin interneuron mean mitolysosome perimeter. Quantitative analysis reveals increased mitolysosome perimeter in geriatric mice compared to young. One-way ANOVA with Bonferroni post hoc. ****$P < 0.0001$, **$P = 0.0015$. $n = 47$. (**R**) CA1 Parvalbumin interneuron mean autolysosome perimeter. Quantitative analysis reveals no alterations in autolysosome perimeter between geriatric mice and young. One-way ANOVA with Bonferroni post hoc. ns = not significant; P value > 0.9999. $n = 27$. (**S**) Dentate gyrus Parvalbumin interneuron mean mitolysosome perimeter. Quantitative analysis reveals increased mitolysosome perimeter in geriatric mice compared to young. One-way ANOVA with Bonferroni post hoc. ****$P < 0.0001$, **$P = 0.0014$. $n = 46$. (**T**) Dentate gyrus Parvalbumin interneuron mean autolysosome perimeter. Quantitative analysis reveals no alterations in autolysosome perimeter between geriatric mice and young. One-way ANOVA with Bonferroni post hoc. ns = not significant; $P > 0.9999$. $n = 27$. (**U**) A9 DA neuron mean mitolysosome perimeter. Quantitative analysis reveals increased mitolysosome perimeter in geriatric mice compared to young. One-way ANOVA with Bonferroni post hoc. ****$P < 0.0001$. $n = 43$. (**V**) A9 DA neuron mean autolysosome perimeter. Quantitative analysis reveals no significant changes in autolysosome perimeter between geriatric mice compared to young. One-way ANOVA with Bonferroni post hoc. ns = not significant; $P > 0.9999$. $n = 19$. (**W**) A10 DA neuron mean mitolysosome perimeter. Quantitative analysis reveals increased mitolysosome perimeter in geriatric mice compared to young. One-way ANOVA with Bonferroni post hoc. ****$P < 0.0001$; ***$P = 0.0001$. $n = 44$. (**X**) A10 DA neuron mean autolysosome perimeter. Quantitative analysis reveals no significant changes in autolysosome perimeter between geriatric mice compared to young. One-way ANOVA with Bonferroni post hoc. ns = not significant; $P = 0.1899$. $n = 26$. (**Y**) Cerebellar astrocyte mean mitolysosome perimeter. Quantitative analysis reveals increased mitolysosome perimeter in geriatric mice compared to young. One-way ANOVA with Bonferroni post hoc. ****$P < 0.0001$. ns = not significant; $P = 0.9203$. $n = 46$. (**Z**) Cerebellar astrocyte mean autolysosome perimeter. Quantitative analysis reveals no significant changes in autolysosome perimeter between geriatric mice compared to young. One-way ANOVA with Bonferroni post hoc. ns = not significant; $P > 0.9999$. $n = 27$. (**AA**) Dentate gyrus astrocyte mean mitolysosome perimeter. Quantitative analysis reveals increased mitolysosome perimeter in geriatric mice compared to young. One-way ANOVA with Bonferroni post hoc. ****$P < 0.0001$. *$P = 0.0165$. $n = 47$. (**AB**) Dentate gyrus astrocyte mean autolysosome perimeter. Quantitative analysis reveals a modest increase in perimeter at midlife compared to young and returning to no significant change in geriatric mice. One-way ANOVA with Bonferroni post hoc. *$P = 0.0240$. ns = not significant; $P = 0.1955$. $n = 27$. (**AC**) Cerebellar microglia mean mitolysosome perimeter. Quantitative analysis reveals increased mitolysosome perimeter in geriatric mice compared to young. One-way ANOVA with Bonferroni post hoc. ****$P < 0.0001$. $n = 46$. (**AD**) Cerebellar microglia mean autolysosome perimeter. Quantitative analysis reveals no significant changes in autolysosome perimeter between geriatric mice compared to young. One-way ANOVA with Bonferroni post hoc. ns = not significant; $P > 0.9999$. $n = 27$. (**AE**) Dentate gyrus microglia mean mitolysosome perimeter. Quantitative analysis reveals increased mitolysosome perimeter in geriatric mice compared to young. One-way ANOVA with Bonferroni post hoc. ****$P < 0.0001$ ***$P = 0.0006$. $n = 47$. (**AF**) Dentate gyrus microglia mean autolysosome perimeter. Quantitative analysis reveals a modest increase in autolysosome perimeter in geriatric mice compared to young. One-way ANOVA with Bonferroni post hoc. *$P = 0.00153$. $n = 27$. (**AG**) ChP mean mitolysosome perimeter. Quantitative analysis reveals a modest increase in mitolysosome perimeter in geriatric mice compared to young. Some fluctuation with increased mitolysosome perimeter is observed at 15 months. One-way ANOVA with Bonferroni post hoc. ***$P = 0.0006$, *$P = 0.0118$. $n = 30$. (**AH**) ChP mean autolysosome perimeter. Quantitative analysis reveals no significant changes in autolysosome perimeter between geriatric mice compared to young. One-way ANOVA with Bonferroni post hoc. ns = not significant; $P > 0.9999$. $n = 22$. Box plots extend from the 25th to the 75th percentiles, with a median line positioned inside the box. Whiskers denote the minimum and maximum values. Source data are available online for this figure.

© The Author(s)

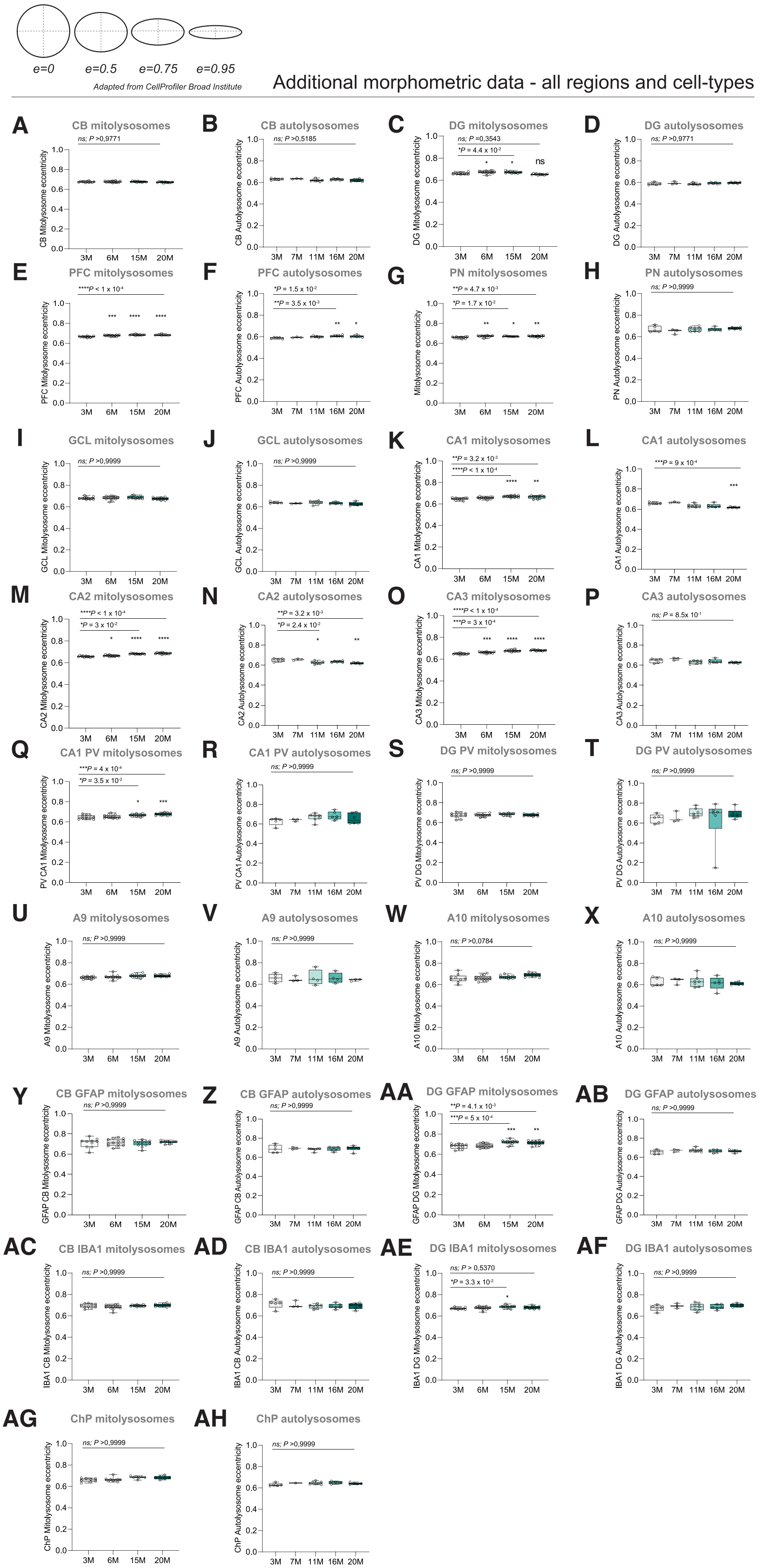
e=0
e=0.5
e=0.75
e=0.95
Adapted from CellProfiler Broad Institute
Additional morphometric data - all regions and cell-types
A CB mitolysosomes
B CB autolysosomes
C DG mitolysosomes
D DG autolysosomes
E PFC mitolysosomes
F PFC autolysosomes
G PN mitolysosomes
H PN autolysosomes
I GCL mitolysosomes
J GCL autolysosomes
K CA1 mitolysosomes
L CA1 autolysosomes
M CA2 mitolysosomes
N CA2 autolysosomes
O CA3 mitolysosomes
P CA3 autolysosomes
Q CA1 PV mitolysosomes
R CA1 PV autolysosomes
S DG PV mitolysosomes
T DG PV autolysosomes
U A9 mitolysosomes
V A9 autolysosomes
W A10 mitolysosomes
X A10 autolysosomes
Y CB GFAP mitolysosomes
Z CB GFAP autolysosomes
AA DG GFAP mitolysosomes
AB DG GFAP autolysosomes
AC CB IBA1 mitolysosomes
AD CB IBA1 autolysosomes
AE DG IBA1 mitolysosomes
AF DG IBA1 autolysosomes
AG ChP mitolysosomes
AH ChP autolysosomes

 © The Author(s)

Figure EV4. Profiling of mitolysosome and autolysosome eccentricity across the aging brain. Contains analysis from all cell-types and regions for completeness.

(**A**) Cerebellar mean mitolysosome eccentricity. Quantitative analysis reveals no significant changes in mitolysosome eccentricity in geriatric mice compared to young. One-way ANOVA with Bonferroni post hoc. ns = not significant; $P > 0.9771$. $n = 47$. (**B**) Cerebellar mean autolysosome eccentricity. Quantitative analysis reveals no significant changes in autolysosome eccentricity between geriatric mice compared to young. One-way ANOVA with Bonferroni post hoc. ns = not significant; $P = 0.5185$. $n = 27$. (**C**) Dentate gyrus mean mitolysosome eccentricity. Quantitative analysis reveals a modest increased mitolysosome eccentricity in during mature adulthood and midlife stages, before returning to corresponding eccentricity values in geriatric animals as in young. One-way ANOVA with Bonferroni post hoc. *$P < 0.0436$. ns = not significant; $P = 0.3543$. $n = 47$. (**D**) Dentate gyrus mean autolysosome eccentricity. Quantitative analysis reveals no significant changes in autolysosome eccentricity between geriatric mice compared to young. One-way ANOVA with Bonferroni post hoc. ns = not significant; $P = 0.9231$. $n = 27$. (**E**) PFC mean mitolysosome eccentricity. Quantitative analysis reveals increased mitolysosome eccentricity in geriatric mice compared to young, indicating an elongated morphology as a function of age. One-way ANOVA with Bonferroni post hoc. ****$P < 0.0001$. $n = 47$. (**F**) PFC mean autolysosome eccentricity. Quantitative analysis reveals a modest increase in autolysosome eccentricity between geriatric mice compared to young animals, indicating mitolysosome elongation. Peak eccentricity values are observed at 16 months. One-way ANOVA with Bonferroni post hoc. *$P = 0.0146$, **$P = 0.0035$. $n = 27$. (**G**) Purkinje cell mean mitolysosome eccentricity. Quantitative analysis reveals increased mitolysosome eccentricity in geriatric mice compared to young. PC mitolysosome undergoes modest fluctuations in eccentricity throughout aging One-way ANOVA with Bonferroni post hoc. *$P = 0.0170$, **$P = 0.0047$. $n = 47$. (**H**) Purkinje cell mean autolysosome eccentricity. Quantitative analysis reveals no significant changes in autolysosome eccentricity between geriatric mice compared to young. One-way ANOVA with Bonferroni post hoc. ns = not significant; $P > 0.9999$. $n = 27$. (**I**) Granular cell layer mean mitolysosome eccentricity. Quantitative analysis reveals no significant changes in mitolysosome eccentricity in geriatric mice compared to young. One-way ANOVA with Bonferroni post hoc. ns = not significant; $P > 0.9999$. $n = 47$. (**J**) Granular cell layer mean autolysosome eccentricity. Quantitative analysis reveals no significant changes in autolysosome eccentricity in geriatric mice compared to young. One-way ANOVA with Bonferroni post hoc. ns = not significant; $P > 0.9999$. $n = 27$. (**K**) CA1 mean mitolysosome eccentricity. Quantitative analysis reveals increased mitolysosome eccentricity in geriatric mice compared to young. One-way ANOVA with Bonferroni post hoc. ****$P < 0.0001$. **$P = 0.0032$. $n = 47$. (**L**) CA1 mean autolysosome eccentricity. Quantitative analysis reveals a significant decline in geriatric mice compared to young, indicating a more elongated shape with aging. One-way ANOVA with Bonferroni post hoc. ***$P = 0.0009$. $n = 27$. (**M**) CA2 mean mitolysosome eccentricity. Quantitative analysis reveals increased mitolysosome eccentricity in geriatric mice compared to young. One-way ANOVA with Bonferroni post hoc. ****P value < 0.0001. *$P = 0.0302$. $n = 47$. (**N**) CA2 mean autolysosome eccentricity. Quantitative analysis reveals a significant decline in geriatric mice compared to young, indicating a more elongated shape with aging. One-way ANOVA with Bonferroni post hoc. **$P = 0.0032$. *$P = 0.0237$. $n = 27$. (**O**) CA3 mean mitolysosome eccentricity. Quantitative analysis reveals increased mitolysosome eccentricity in geriatric mice compared to young. One-way ANOVA with Bonferroni post hoc. ****$P < 0.0001$. ***$P = 0.0003$. $n = 47$. (**P**) CA3 mean autolysosome eccentricity. Quantitative analysis reveals no significant change in geriatric mice compared to young. One-way ANOVA with Bonferroni post hoc. ns = not significant; $P = 0.8531$. $n = 27$. (**Q**) CA1 Parvalbumin interneuron mean mitolysosome eccentricity. Quantitative analysis reveals increased mitolysosome eccentricity in geriatric mice compared to young. One-way ANOVA with Bonferroni post hoc. ***$P = 0.0004$. *$P = 0.0350$. $n = 47$. (**R**) CA1 Parvalbumin interneuron mean autolysosome eccentricity. Quantitative analysis reveals no significant changes in autolysosome eccentricity in geriatric mice compared to young. One-way ANOVA with Bonferroni post hoc. ns = not significant; $P > 0.9999$. $n = 27$. (**S**) Dentate gyrus Parvalbumin interneuron mean mitolysosome eccentricity. Quantitative analysis reveals no significant change in geriatric mice compared to young. One-way ANOVA with Bonferroni post hoc. ns = not significant; $P > 0.9999$. $n = 46$. (**T**) Dentate gyrus Parvalbumin interneuron mean autolysosome eccentricity. Quantitative analysis reveals no significant change in geriatric mice compared to young. One-way ANOVA with Bonferroni post hoc. ns = not significant; $P > 0.9999$. $n = 27$. (**U**) A9 DA neuron mean mitolysosome eccentricity. Quantitative analysis reveals increased mitolysosome eccentricity in geriatric mice compared to young. One-way ANOVA with Bonferroni post hoc. ns = not significant; $P > 0.0689$. $n = 43$. (**V**) A9 DA neuron mean autolysosome eccentricity. Quantitative analysis reveals no significant changes in autolysosome eccentricity between geriatric mice compared to young. One-way ANOVA with Bonferroni post hoc. ns = not significant; $P > 0.9999$. $n = 19$. (**W**) A10 DA neuron mean mitolysosome eccentricity. Quantitative analysis reveals increased mitolysosome eccentricity in geriatric mice compared to young. One-way ANOVA with Bonferroni post hoc. ns = not significant; $P > 0.0784$. $n = 44$. (**X**) A10 DA neuron mean autolysosome eccentricity. Quantitative analysis reveals no significant changes in autolysosome eccentricity between geriatric mice compared to young. One-way ANOVA with Bonferroni post hoc. ns = not significant; $P > 0.9999$. $n = 26$. (**Y**) Cerebellar astrocyte mean mitolysosome eccentricity. Quantitative analysis reveals no significant change in mitolysosome eccentricity in geriatric mice compared to young. One-way ANOVA with Bonferroni post hoc. ns = not significant; $P > 0.9999$. $n = 46$. (**Z**) Cerebellar astrocyte mean autolysosome eccentricity. Quantitative analysis reveals no significant changes in autolysosome eccentricity between geriatric mice compared to young. One-way ANOVA with Bonferroni post hoc. ns = not significant; $P > 0.9999$. $n = 27$. (**AA**) Dentate gyrus astrocyte mean mitolysosome eccentricity. Quantitative analysis reveals increased mitolysosome eccentricity in geriatric mice compared to young animals. One-way ANOVA with Bonferroni post hoc. ***$P = 0.0005$. **$P = 0.0041$. $n = 47$. (**AB**) Dentate gyrus astrocyte mean autolysosome eccentricity. Quantitative analysis reveals no significant changes between geriatric mice compared to young. One-way ANOVA with Bonferroni post hoc. ns = not significant; P value > 0.9999. $n = 27$. (**AC**) Cerebellar microglia mean mitolysosome eccentricity. Quantitative analysis reveals increased mitolysosome eccentricity in geriatric mice compared to young. One-way ANOVA with Bonferroni post hoc. ns = not significant; $P > 0.7837$. $n = 46$. (**AD**) Cerebellar microglia mean autolysosome eccentricity. Quantitative analysis reveals no significant changes in autolysosome eccentricity between geriatric mice compared to young. One-way ANOVA with Bonferroni post hoc. ns = not significant; $P > 0.9999$. $n = 27$. (**AE**) Dentate gyrus microglia mean mitolysosome eccentricity. Quantitative analysis reveals no significant change in mitolysosome eccentricity in geriatric mice compared to young animals, although a modest increase can be observed at 15 months One-way ANOVA with Bonferroni post hoc. *$P = 0.0328$, ns = not significant; $P > 0.5370$. $n = 47$. (**AF**) Dentate gyrus microglia mean autolysosome eccentricity. Quantitative analysis reveals no significant changes in autolysosome eccentricity between geriatric mice compared to young. One-way ANOVA with Bonferroni post hoc. ns = not significant; $P > 0.9999$. $n = 27$. (**AG**) ChP mean mitolysosome eccentricity. Quantitative analysis reveals increased mitolysosome eccentricity in geriatric mice compared to young. One-way ANOVA with Bonferroni post hoc. ns = not significant; $P = 0.0922$. $n = 30$. (**AH**) ChP mean autolysosome eccentricity. Quantitative analysis reveals no significant changes in autolysosome eccentricity between geriatric mice compared to young. One-way ANOVA with Bonferroni post hoc. ns = not significant $P > 0.9999$. $n = 22$. Box plots extend from the 25th to the 75th percentiles, with a median line positioned inside the box. Whiskers denote the minimum and maximum values. Source data are available online for this figure.

© The Author(s)

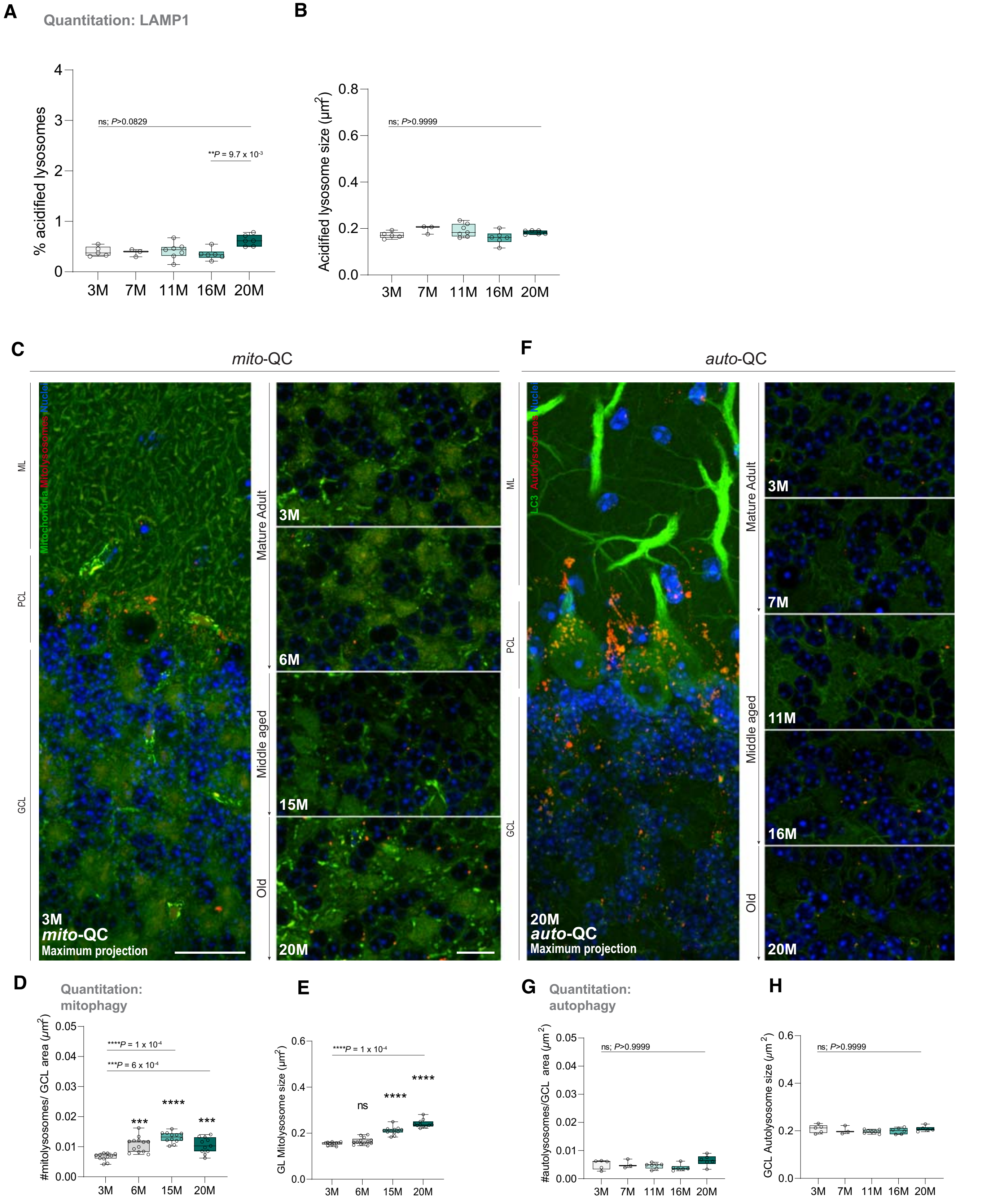
A
Quantitation: LAMP1
% acidified lysosomes
ns; P>0.0829
**P = 9.7 x 10-3
3M 7M 11M 16M 20M
B
Acidified lysosome size (µm2)
ns; P>0.9999
3M 7M 11M 16M 20M
C
mito-QC
Mitochondria Mitolysosomes Nuclei
ML
PCL
GCL
3M
mito-QC
Maximum projection
Mature Adult
Middle aged
Old
3M
6M
15M
20M
F
auto-QC
LC3 Autolysosomes Nuclei
20M
auto-QC
Maximum projection
7M
11M
16M
D
Quantitation: mitophagy
#mitolysosomes/ GCL area (µm2)
****P = 1 x 10-4
***P = 6 x 10-4
E
GL Mitolysosome size (µm2)
ns
G
Quantitation: autophagy
#autolysosomes/GCL area (µm2)
H
GCL Autolysosome size (µm2)

 © The Author(s)

Figure EV5. Mitophagy and macroautophagy in cerebellar granule neurons in vivo.

(**A**) Purkinje cell acidified autolysosomes. The number of LAMP1-positive structures that are double-positive for mCherry-only remains unchanged between young and geriatric mice. Some fluctuations of increased acidified autolysosomes can be observed at 16 months compared to the geriatric. One-way ANOVA with Bonferroni post hoc. $^{**}P < 0.0097$, ns = not significant; $P > 0.0829$. $n = 27$. (**B**) Purkinje cell mean acidified autolysosome size. mCherry-LAMP1 double-positive structure size remain constant throughout aging. One-way ANOVA with Bonferroni post hoc. ns = not significant; $P > 0.9999$. $n = 27$. (**C–E**) Mitophagy in the aging GCL. Representative images of mitophagy events throughout aging in the *mito*-QC granular cell layer. Quantitative analysis reveals increased levels of mitophagy in geriatric mice compared to young, as do mean mitolysosome size. One-way ANOVA with Bonferroni post hoc. Scale bars = 20 µm. $^{****}P < 0.0001$. $n = 47$. (**F–H**) Autophagy in the aging GCL. Representative images of autophagy events throughout aging in the *auto*-QC granular cell layer. Quantitative analysis reveals unaltered levels of autophagy in geriatric mice compared to young, nor do autolysosome size change. One-way ANOVA with Bonferroni post hoc. Scale bars = 20 µm. ns = not significant; $P > 0.9999$. $n = 27$. Box plots extend from the 25th to the 75th percentiles, with a median line positioned inside the box. Whiskers denote the minimum and maximum values. Source data are available online for this figure.

© The Author(s)
